# Reaction-Agnostic Featurization of Bidentate Ligands for Bayesian Ridge Regression of Enantioselectivity

**DOI:** 10.1021/acscatal.4c02452

**Published:** 2024-06-04

**Authors:** Alexandre A. Schoepfer, Ruben Laplaza, Matthew D. Wodrich, Jerome Waser, Clemence Corminboeuf

**Affiliations:** † Laboratory for Computational Molecular Design, Institute of Chemical Sciences and Engineering, École Polytechnique Fédérale de Lausanne (EPFL), 1015 Lausanne, Switzerland; ‡ Laboratory of Catalysis and Organic Synthesis, Institute of Chemical Sciences and Engineering, École Polytechnique Fédérale de Lausanne (EPFL), 1015 Lausanne, Switzerland; § National Center for Competence in Research-Catalysis (NCCR-Catalysis), École Polytechnique Fédérale de Lausanne, 1015 Lausanne, Switzerland

**Keywords:** homogeneous catalysis, bidentate ligands, asymmetric catalysis, Bayesian optimization, machine learning

## Abstract

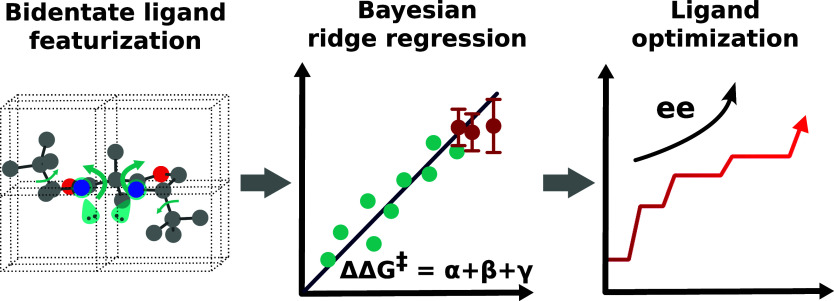

Chiral ligands are important components in asymmetric homogeneous catalysis, but their synthesis and screening can be both time-consuming and resource-intensive. Data-driven approaches, in contrast to screening procedures based on intuition, have the potential to reduce the time and resources needed for reaction optimization by more rapidly identifying an ideal catalyst. These approaches, however, are often nontransferable and cannot be applied across different reactions. To overcome this drawback, we introduce a general featurization strategy for bidentate ligands that is coupled with an automated feature selection pipeline and Bayesian ridge regression to perform multivariate linear regression modeling. This approach, which is applicable to any reaction, incorporates electronic, steric, and topological features (rigidity/flexibility, branching, geometry, and constitution) and is well-suited for early stage ligand optimization. Using only small data sets, our workflow capably predicts the enantioselectivity of four metal-catalyzed asymmetric reactions. Uncertainty estimates provided by Bayesian ridge regression permit the use of Bayesian optimization to efficiently explore pools of prospective ligands. Finally, we constructed the BDL-Cu-2023 data set, composed of 312 bidentate ligands extracted from the Cambridge Structural Database, and screened it with this procedure to identify ligand candidates for a challenging asymmetric oxy-alkynylation reaction.

## 1 Introduction

Statistical methods accelerate the discovery and optimization of chemical reactions in homogeneous catalysis.^1−18^ Employing these “data-driven” approaches requires abundant, high-quality data^19−21^ that are often scarce. Ligand optimization, in particular, suffers from this problem since most experimental data sets tend to be size-limited as a result of ligand screening campaigns that often consist of fewer than a dozen experiments. In such “low data” regimes, nonlinear statistical models perform poorly due to overfitting. On the other hand, multivariate linear regression (MLR) models offer data-efficient and intuitive alternatives that can be developed from only a few samples yet are robust, interpretative (i.e., the way they work is exposed and understandable), and often extrapolative to unseen ligands.

To develop MLR models, catalysts are usually first optimized using density functional theory (DFT) and then featurized.^13,16,17,22−31^ The resulting molecular features (e.g., atomic charges, local stretching frequencies, and cone/bite angles) are low-dimensional and highly interpretable,^17,32−34^ which allows design principles and hypothesized reaction pathways to be derived from the fitted models.^18,22,23,26,28−31,35^ This established approach, however, suffers from two significant drawbacks: first, specific features for the chemical problem of interest must be selected for the MLR model and second, only the most relevant of these features are used in developing the final model. As a result, MLRs are often not transferable to different settings (e.g., a similar reaction incorporating a different family of ligands) as those features previously selected may not be defined. To avoid this issue, molecular grids where similar structures are superimposed have been used.^2,22−24,36−40^ Alternatively, Gensch et al.^41^ recently introduced a comprehensive featurization strategy for monodentate organophosphorus ligands that facilitates the creation of MLRs for any possible reaction class. Establishing this paradigm for more complex ligand types is of great interest for developing transferable predictive models across catalyst families and reaction classes.^17^

To this end, here we present a reaction-agnostic workflow applied to bidentate ligands that employs, among others, rarely used topological-based features.^3,42−44^ Coupling this featurization strategy with an automated feature selection pipeline using Bayesian ridge regression (BRR)^45^ allows development of models that capably predict the enantioselectivity of four different reaction classes while highlighting the importance of using topological-based features. Moreover, by leveraging the calibrated uncertainty estimations from BRR, we demonstrate Bayesian optimization (BO) for optimal ligand screening^46−53^ on the BDL-Cu-2023 data set, an original pool of 312 chiral bidentate ligands extracted from the Cambridge Structural Database (CSD). Previous work using BO for catalyst optimization used either Gaussian process regression (GPR) or ensembles of models.^54,55^ Overall, this work demonstrates that linear models are more accurate and data-efficient than nonlinear methods in the “extremely low” data regime.

## 2 Methods

### 2.1 Data Sets

**Scheme 1:**
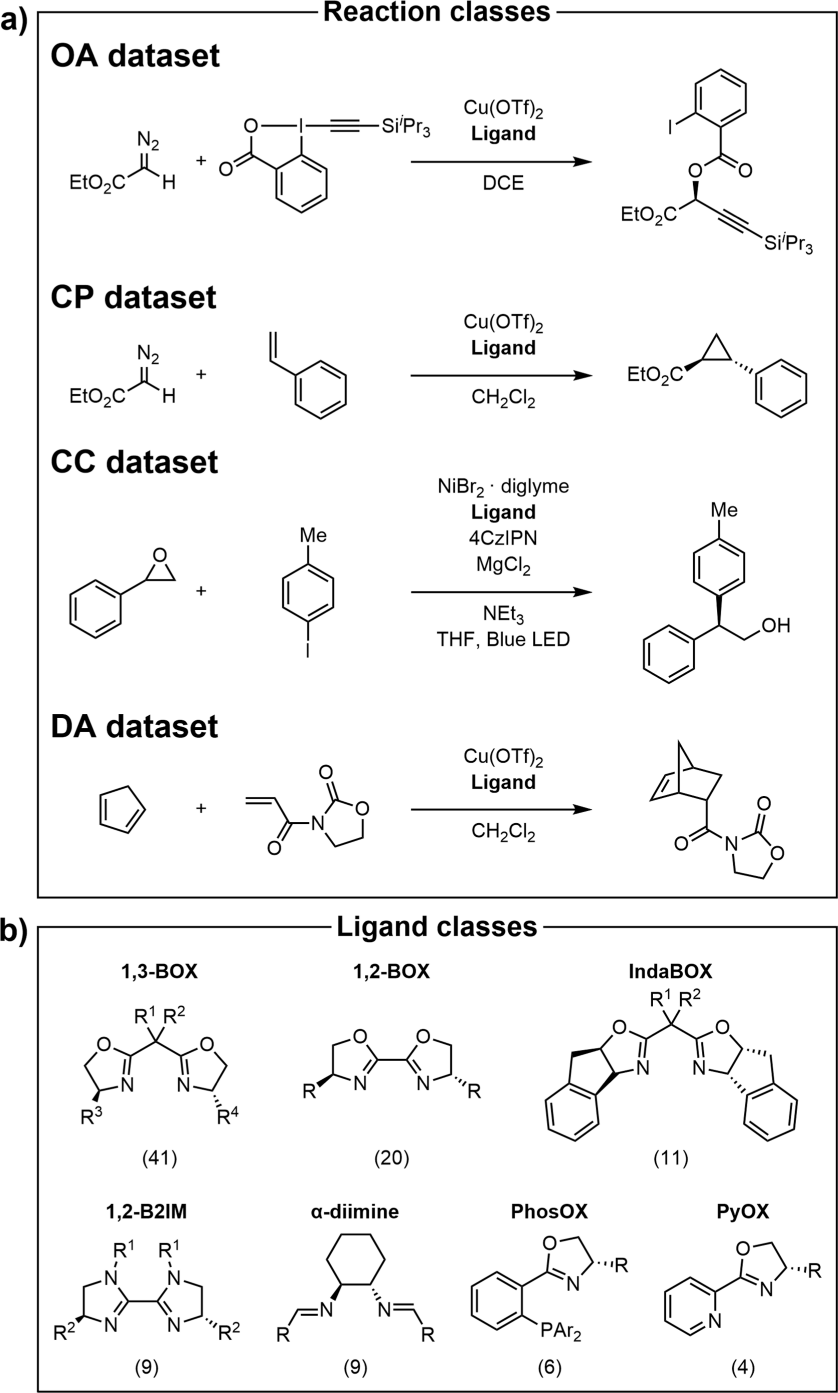
(a) Candidate Asymmetric Reactions Used to Develop and Test the Presented Pipeline; (b) Ligand Families Used in These Reaction Classes The number of unique ligands per class is shown in parentheses.

To develop, train, and test our pipeline, four asymmetric reaction classes that previously underwent extensive experimental ligand screening were selected for examination (Scheme 1, top): copper-catalyzed oxy-alkynylation of diazo compounds with hypervalent iodine reagents (**OA**),^56^ copper-catalyzed cyclopropanation of styrene with diazo esters (**CP**),^57^ nickel/photoredox-catalyzed cross-electrophile coupling of styrene oxides with aryl iodides (**CC**),^26^ and a copper-catalyzed Diels–Alder ligand benchmark reaction with cyclopentadiene and an imide (**DA**).^58−64^Table 1 gives an overview of these data sets. For each reaction, the reactants, reagents (except the ligand), and solvent were kept constant, while reaction conditions (metal loading, time, temperature, etc.) varied among experiments. For this reason, the data sets are small (ranging from 19 to 30 data points) as they include only ligand screening experiments (see Supporting Information Section S2 for details). Our approach currently does not account for substrate effects, rather we choose to focus on catalyst optimization (see Supporting Information Section S5.1 for discussion). For three of the four curated data sets, all ligands originated from a single publication, while the fourth data set (**DA**) contains ligands taken from seven different publications, which introduces additional noise in the data resulting from different experimental setups. For the **OA** data set, additional ligands with enantiomeric excesses not part of the original publication^56^ were included from electronic laboratory notebook entries (see Supporting Information Section S2.1).

**Table 1:** Overview of the Data Sets Used in This Work

data set	**OA**	**CP**	**CC**	**DA**
# of data points	19	30	29	30
# of publications	1	1	1	7
oxazoline ligands	16	30	20	21

Combining all four data sets gives a total of 100 unique bidentate ligands, which were curated as a ligand pool for exploration (see Section 3.2). Most reactions (across all data sets) used bis-oxazoline (BOX)-type ligands (see Table 1), but other ligand classes [bi-2-imidazolines (B2IM), α-diimines, phosphorus-oxazolines (PhosOX), and pyridine-oxazolines (PyOX, Scheme 1, bottom)] are also present in smaller numbers. In addition, bidentate ligands bound to Cu(I) or Cu(II) found in the CSD^65,66^ were extracted with cell2mol .^67^ This yielded 312 new ligands possessing at least one chiral atom, the most common being N,N- and O,N-ligands (P,N-, P,O-, P,P-, S,N-, S,O-, and S,S-ligands are also present although in smaller numbers, see Supporting Information Section S2.2). This separate pool, which we call the BDL-Cu-2023 data set, was used to conduct further exploration (vide infra).

### 2.2 Bidentate Ligand Featurization

**Figure 1:**
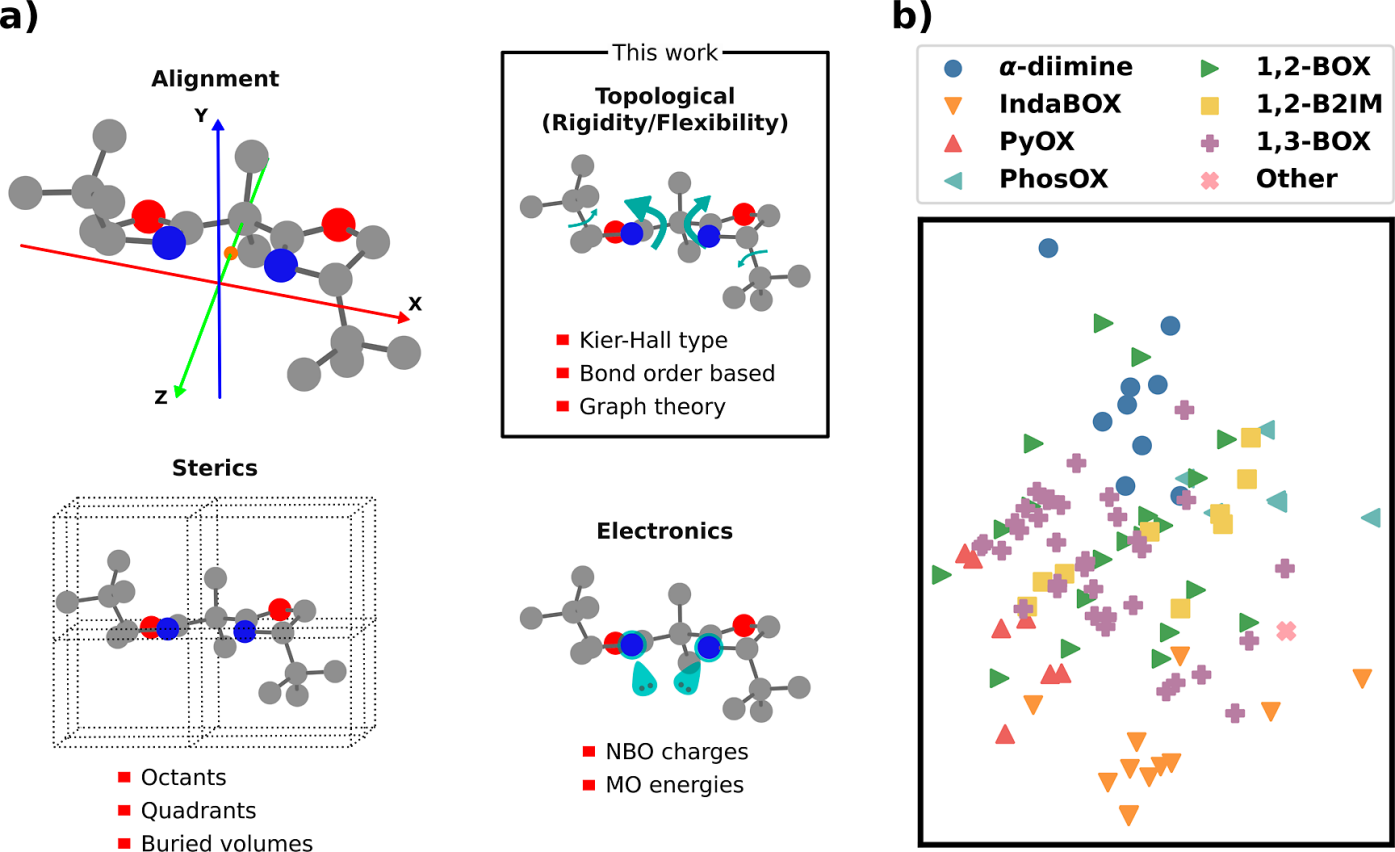
Featurization of bidentate ligands. (a) Alignment of the bidentate ligands in space and feature classes. (b) PCA map of the feature space for all ligands with all available features.

Molecular features (i.e., molecular descriptors) were split into three categories: electronic, steric, and topological (Figure 1a) and further categorized based on their intensive or extensive nature. To maximize generality, the only local features used describe the ligand’s two complexing atoms (that bind the metal), which are present in all bidentate ligands. Steric features were computed using a consistent alignment for all ligands that allows the ligand’s molecular volume to be reproducibly split into octants, quadrants, and halves (see Supporting Information Section S4.1). Full and buried volumes were computed following the recommendations of Cavallo et al.^68^

Topological features, seldom exploited in multilinear regression models for homogeneous catalysis, were determined from vertex and edge information on the ligand’s molecular graph that were generated using covalent radii to assign bonds based on the DFT-optimized geometry. This category includes global topological features (e.g., Wiener, Hosoya *Z*, and Balaban *J* indices),^69−71^ bond-fragment-based descriptors (e.g., the indices introduced by Kier and Hall),^72−79^ and bond-order quantities (e.g., local and global simple indices and the CREST flexibility index).^80,81^ Variants of existing topological descriptors, originally used for drug design, were also developed and included to capture catalyst rigidity/flexibility.

Summed together, this strategy yields a total of 232 features for each ligand, which constitutes a representation of bidentate ligand space. The principal component analysis (PCA) plot of the featurized ligands in Figure 1b showcases how these features assemble ligands belonging to the same family (see Scheme 1b) while keeping related ligand families adjacent, in agreement with chemical intuition. Note that, by design, all features can be obtained for any possible bidentate ligand using the Moltop package and associated scripts available at https://github.com/lcmd-epfl/rafbl. Further details and a complete description of all 232 features are given in the Supporting Information (Section S4).

### 2.3 Regression and Optimization Methods

In this work, BRR (a regularized variation of least-squares fitting) was used to fit the MLR models whose parameters were estimated using Bayesian inference, which provides a calibrated uncertainty for each prediction. To avoid overfitting, model complexity was limited to a maximum of three features per model using a forward-step feature selection technique (see Supporting Information Section S5.2 for details).^82^ We find that this approach leads to highly interpretable, robust models that outperform nonlinear models (see Supporting Information Section S5.5 for a detailed comparison).

To guide ligand screening, a pool-based BO, in which prospective ligands are run through the BRR-fitted model, was employed. For each ligand **x** in the pool, the expected improvement (EI)^83^ defined as
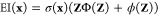
1
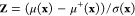
2was computed. Here, μ represents the predicted value, μ^+^ the current best value, σ the standard deviation, Φ the cumulative distribution function, and ϕ the probability density function. New results are subsequently incorporated into the training set, and the process is repeated until unexplored ligands each have EI scores lower than those already seen by the model. This implies that no further improvement (i.e., no better ligand) is expected within the pool.

### 2.4 General Workflow

**Figure 2:**
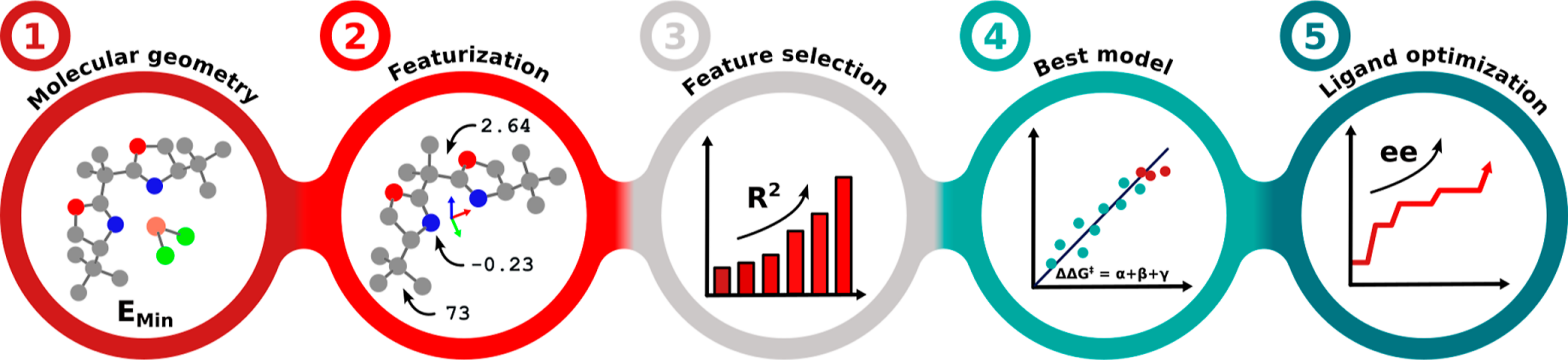
General workflow for model selection. (1) Ligands are optimized with a metal center to obtain the desired geometry. (2) 232 features are extracted from the metal-free structure. (3) The most promising feature combinations are identified through testing. (4) The best features undergo cross-validation, and the best BRR model is obtained. (5) This resulting model is used for ligand screening with BO.

Figure 2 provides an overview of our proposed workflow. In step 1, CuCl_2_L structures are optimized at the PBE0-D3(BJ)/def2-SVP level (see Computational Details), followed by automatic feature extraction for each structure (Step 2). The Eyring equation is then used to convert enantiomeric excesses (for experimentally available data) to energies (ΔΔ*G*^⧧^) with the corresponding reaction temperature. Using the forward step technique in combination with BRR, the most promising feature combinations are selected (Step 3, see Section 2.3). The initial model selection is then evaluated with leave-one-out cross validation (LOO), and the most promising model is chosen using the adjusted *R*^2^ of the left-out samples (adj. *R*_LOO_^2^, Step 4, see Supporting Information Section S5.2). With the final model, a pool of ligands is screened, and BO is used to identify the most promising candidates, which should next be tested (Step 5).

## 3 Results and Discussion

### 3.1 Generation of MLR Models

**Figure 3:**
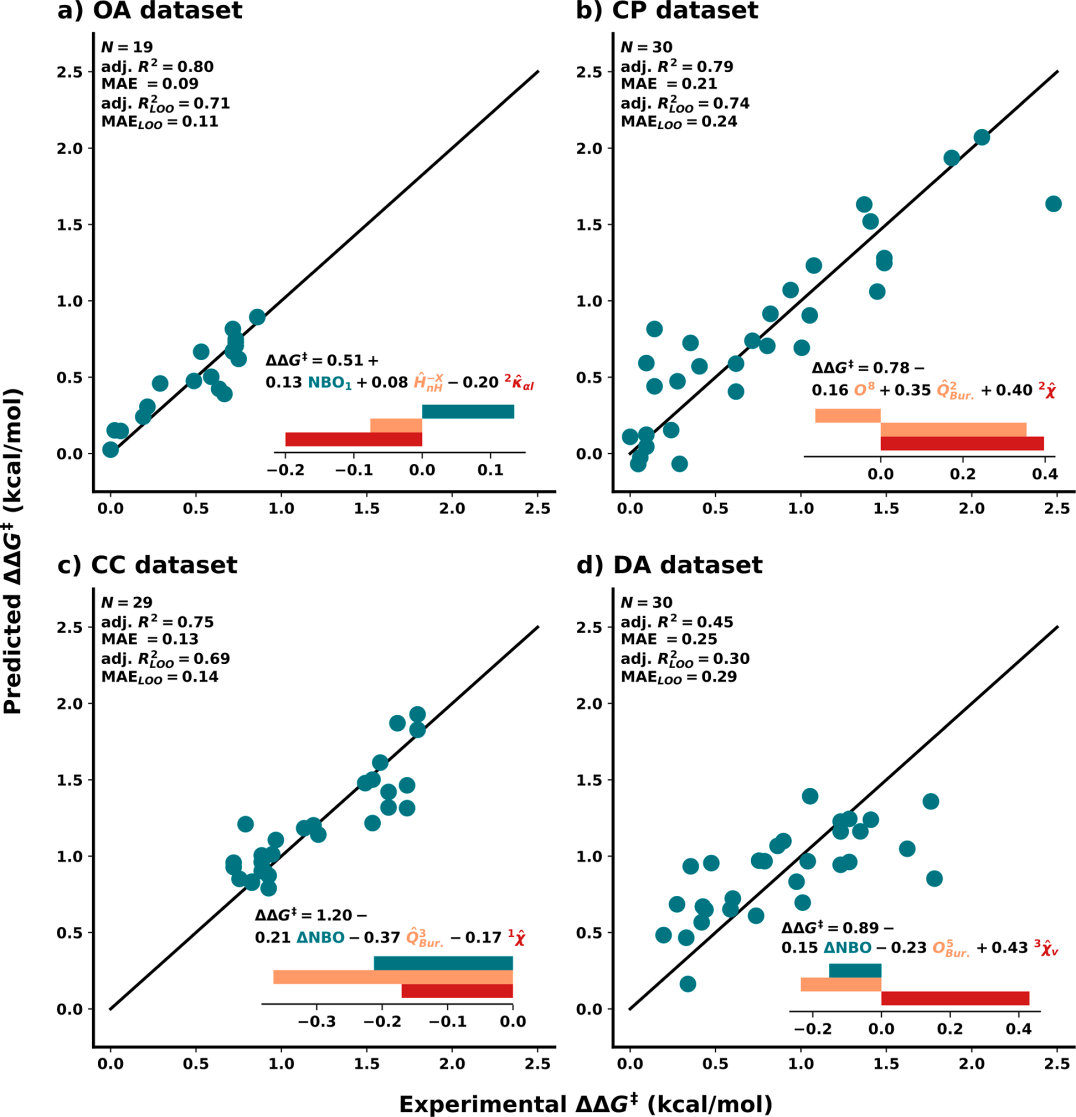
Fitted models for the four data sets, where *N* is the number of points, adj. *R*^2^ is the adjusted coefficient of determination, MAE is the mean absolute error, and the LOO subscript indicates the scores for the leave-one-out cross-validation. The model equation is represented in each plot with a depiction of the normalized weights. Electronic features are represented in teal, steric in orange, and topological in red. (a, **OA** data set) Lone-pair NBO energies of the smaller half, normalized hydrogen-free volume of the smaller half (−*x*), and normalized original Kier ^2^κ_α*l*_ index. (b, **CP** data set) Volume of octant 8 (*x*, *y*, −*z*), buried volume of the northwest quadrant *Q*^2^ (−*x*, *y*), and normalized ^2^χ. (c, **CC** data set) Absolute difference of lone-pair NBO energies, normalized buried volume of the southwest quadrant *Q*^3^ (−*x*, −*y*), and normalized ^1^χ index. (d, **DA** data set) Absolute difference of lone-pair NBO energies, buried volume of octant 5 (*x*, −*y*, −*z*), and normalized ^3^χ_*v*_ index. See Supporting Information Section S4 for a complete description of all features.

Using the above pipeline, an interpretable MLR model was generated for each of the four Scheme 1 data sets (Figure 3). Recall that no human input was required for featurization, feature selection, and generation of the final models (i.e., all reactions used the same initial features and were run through the pipeline in an automated fashion). In general, the models perform well, with MAE_LOO_ not being higher than 0.29 kcal/mol. As in standard MLR, examining the normalized weights of these models reveals insight into the key aspects of the ligand that lead to high enantioselectivity (Figure 3).

For the oxy-alkynylation of diazo compounds model (**OA**, Figure 3a), the selected features are the lone-pair natural bond orbital (NBO) energies of the smaller ligand half (−*x*, electronic), the normalized hydrogen-free volume of the smaller half (−*x*, steric), and the normalized original Kier ^2^κ_α*l*_ index (lower values—more rigid, topological). The large topological feature weight indicates that a rigid catalyst structure is the most crucial element in determining enantioselectivity with sterics and electronics relegated to smaller roles. Taken together, these design principles indicate that indane-derived BOX (IndaBOX) ligands are well suited for this reaction as they are simultaneously both bulky and rigid. We hypothesize that catalyst rigidity contributes to a greater difference between the energies of the transition states leading to both enantiomers of the product.

For the cyclopropanation of styrene with diazo esters model (**CP**, Figure 3b), no electronic feature but rather two steric features were selected: an octant (*x*, *y*, −*z*, steric), the normalized northwest quadrant (−*x*, *y*, steric), and the normalized Hall ^2^χ index (higher values—more rigid, topological). Combined, sterics play a more important role than rigidity, in agreement with previously proposed models.^57^ A closer examination of the model reveals that the −*x*, *y* quadrant  is especially important for enantioselectivity. Nevertheless, the Hall index possesses the highest weight overall, and its importance should thus not be ignored.

For the cross-electrophile coupling of styrene oxides with aryl iodides model (**CC**, Figure 3c), the selected features are the difference of lone-pair NBO energies (electronic), the normalized southwest quadrant (−*x*, −*y*, steric), and the normalized ^1^χ Hall index (higher values—more bonded, topological). Here, the most important feature is steric  and indicates that the southwest quadrant should be kept free, which aligns with previous postulations regarding the origin of stereoselectivity.^26^ Overall, the family of B2IM ligands with closed, more branched backbones matches well with the features of the model. Note that the adjusted *R*^2^ of 0.75 of our model is similar to the adjusted *R*^2^ of 0.74 previously reported for **CC**,^26^ which shows that our pipeline yields similar predictivity and analogous interpretation without requiring information about reaction intermediates.

Finally, for the Diels–Alder reaction of cyclopentadiene in an imide model (**DA**, Figure 3d), the selected features are the NBO energies (electronic), a buried hydrogen-free octant (*x*, −*y*, −*z*, steric), and the normalized ^3^χ_*v*_ Hall index (higher values—more rigid, topological). Here, as in the **OA** data set, the topological feature is found to be dominant. Being built from seven different publications, this reaction is particularly challenging, and the reported adjusted *R*^2^ is rather poor. Nevertheless, our model has cross-validated errors of less than 0.3 kcal/mol.

The MLR models obtained from our pipeline for each of the four reaction data sets discussed above are both simple and interpretable owing to their limited number of features and selected composition. The importance of topological features that describe catalyst rigidity/flexibility (which, recall, are typically absent in multilinear regression models for homogeneous catalysis) across all four reactions is noteworthy as in three out of four cases these factors play the most important role (as seen through examination of the normalized weights).

### 3.2 Pool-Based Ligand Optimization with BRR and BO

**Figure 4:**
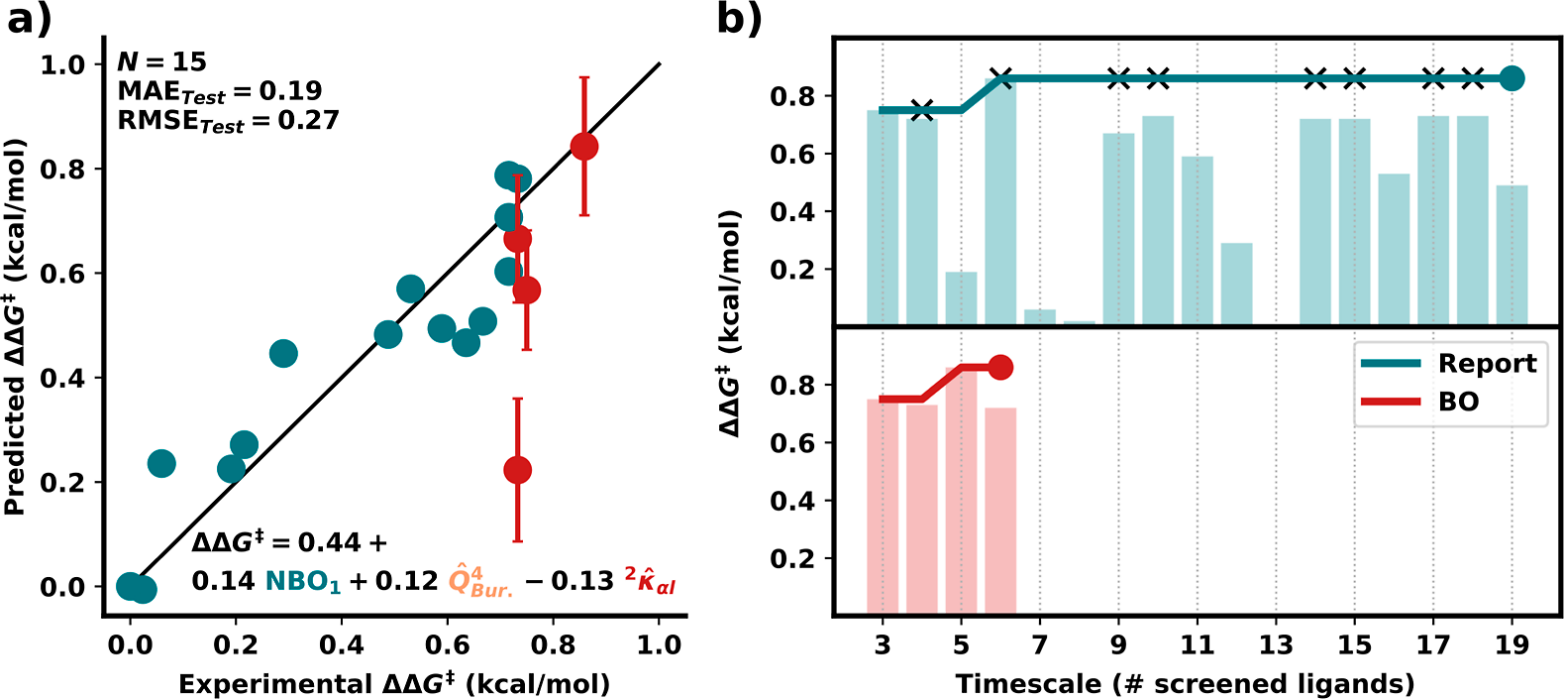
Retrospective and prospective experiments for the **OA** data set. (a) 80/20 train (teal)/test (red) split. Error bars correspond to 1σ. The selected features are lone-pair NBO energies of the smaller half, buried volume of the southeast quadrant *Q*^4^ (*x*, −*y*), and the normalized original ^2^κ_α*l*_. (b) Retrospective BO analysis. The “Report” line follows the original optimization timeline. New batches of ligands are represented as black crosses. Bar plots represent the ΔΔ*G*^⧧^ values of each ligand in the batches. The BO starts with the three points from the first batch.

As illustrated above, the fitted BRR models can be used to elucidate design principles by analyzing the selected features/weights and interpreting the trends. Additionally, they may also be directly employed for ligand optimization (e.g., to predict ligands that will impart higher selectivity). In this context, the ability to accurately predict higher ΔΔ*G*^⧧^ values than those present in the data set in unseen samples is crucial, particularly for cases where the training set contains only ligands with low enantioselectivities (e.g., an initial batch based on similar reactions). To simulate this situation, we performed an 80/20 train/test split on the **OA** data set to test the model on out-of-range predictions (see Supporting Information Section S5.4 for more tests). As shown in Figure 4a, the test set includes the four best experimental ligands (red points), while the training set contains ligands with similar or worse performance (teal points). The complete pipeline was then rerun using the reduced training data (teal points alone), which produced a similar (but not identical) model to that shown in Figure 3. Overall, this refit model shows low errors (MAE of 0.19 kcal/mol) for unseen samples and well-calibrated uncertainties that are nearly within 1σ from the reported experimental values except for one outlier (BOX ligand with an additional oxazoline arm). Comparing this model with the full model (Figure 3a), it is easy to spot that this inaccuracy is caused by the steric feature. The enhanced uncertainty estimation, powered by BRR, coupled with the low prediction error on the test set, demonstrates that our pipeline yields models capable of accurately predicting the selectivity of unknown ligands, including those anticipated to have greater selectivity than that of the training set.

Ideally, the newly developed BRR model can be used to more rapidly identify an ideal catalyst, which would avoid performing experiments that yield no improvement past the optimum. To assess this, we used the dates reported in the original electronic laboratory notebook to construct a timeline depicting the experimental reporting of each ligand (teal, Figure 4b). Here, the best-performing ligand was found between the sixth and eighth experiments; all subsequent attempts did not yield any further improvement. Having established the model’s ability to reliably predict out-of-range ligands with calibrated uncertainties (vide supra), we conducted BO to efficiently find the optimal ligand from a pool of candidates using the same initial three ligands as the training set. As shown in red (Figure 4b), the acquisition function identifies the best ligand as the second candidate to be tested (fifth total ligand, including the three included in the initial training set), faster than the original experimental optimization procedure. From that point forward, one additional ligand is (incorrectly) predicted to bring potential improvement; however, given the significant amount of uncertainty, the additional specimen ultimately did not demonstrate improved selectivity. At this point, the stop criterion was met as no other ligand in the pool was predicted to provide further improvement, in agreement with experimental observations.

Compared to the original purely experimental screening, using the BO pipeline reduced the total number of reactions performed by a factor of 3 (6 vs 19). Thus, BO succeeds, even in the low-data regime, at rapidly identifying the best ligand from the candidate pool while avoiding wasteful experiments.

**Figure 5:**
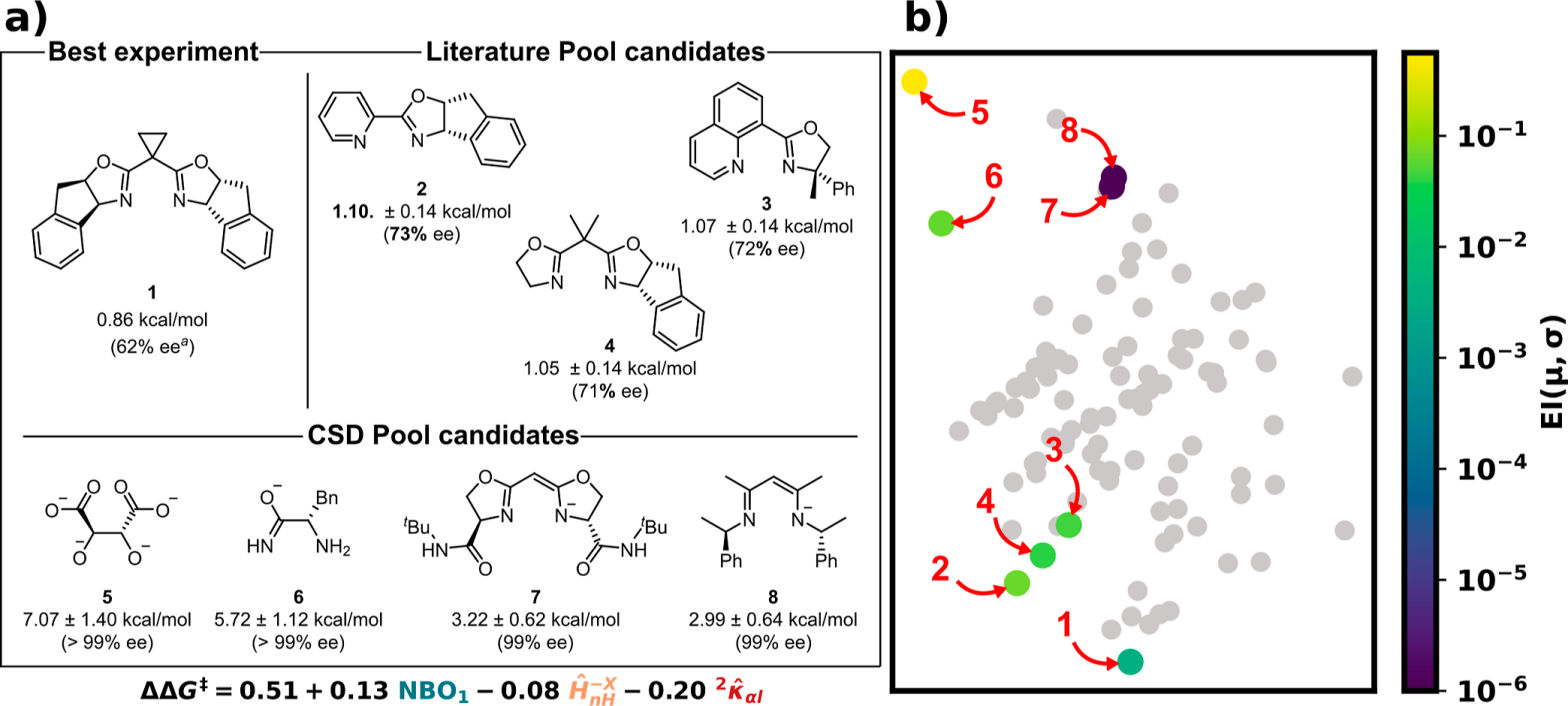
Predictions and analysis of literature and CSD-extracted ligands for the **OA** reaction. (a) Top left: the best ligand found experimentally. Top right: the top three EI candidates from pool-based predictions for the reaction using the ligands from the three remaining literature data sets. Bottom: four diverse candidate ligands from the CSD which exhibit a high EI score. The predicted ΔΔ*G*^⧧^ and its uncertainty are given. The used model is given at the bottom. (b) PCA map of the candidate ligands using the same embedding as in Figure 1b (gray points). The coloring corresponds to EI scores (truncated to 10^–6^ for clarity). Red numbers correspond to structures in panel (a). ^a^Optimized reaction conditions with this ligand yield 90% ee.

With these promising results in hand, we screened ligands reported in the **CP**, **CC**, and **DA** reactions (Scheme 1) to test their enantioselectivity for the **OA** reaction. Figure 5a (top left) shows the best ligand found experimentally, as well as the top three “not-yet-sampled” ligand candidates derived from the other three reaction classes that were predicted by the BRR. As each of these ligand structures closely matches those previously tested experimentally, unsurprisingly, ligands (**2**–**4**) are predicted to yield only minimal improvement over the current best ligand (**1**) from the **OA** set. Since these “Literature Pool candidate” ligands are so structurally similar to those found in the **OA** pool, their experimental testing corresponds to a “low-risk, low-reward” situation, where the model is reliable, but potential improvements in selectivity are anticipated to be minor.

On the other hand, searching for a much broader range of structures, such as the 312 bidentate chiral ligands extracted from the CSD (see Section 2.1), constitutes a much different approach. In this “high-risk, high-reward” situation, the model is highly prone to errors (due to having never seen these types of structures) but can serve as a means to move toward new regions of ligand space that the model finds promising. As an example, four newly identified ligand classes (**5**–**8**) each possessing distinct electronic properties (i.e., negative charges) from the original sets of tested ligands, as well as high rigidities (for **7** and **8**), are shown in Figure 5a “CSD Pool candidates”. Here, the presence of oxygen- and electron-rich nitrogen atoms imparts substantially higher NBO charges than those of the previously tested ligands, which leads to higher predicted enantioselectivities (along with higher predicted uncertainties!) according to the linear model.

To assess this increase in diversity, these “high-risk, high-reward” CSD ligands were plotted with the previously discussed dimensionality reduction embedding (Figure 5b). In general, identified literature-extracted ligands (**1**–**4**) are found close to the bottom of the map. **7** and **8** are situated near the α-diimine and 1,2-BOX regions as they most closely resemble these classes. On the other hand, the most promising candidates (with high EI scores) **5** and **6**, which belong to new classes of ligands within the CSD set, were unexplored in the experimental ligand screenings.

## 4 Conclusions

In this work, we introduced a general workflow for constructing linear models from small numbers of screening experiments that predict enantioselectivity in reactions involving bidentate ligands. Data sets comprising four different reaction classes (totaling 100 bidentate ligands belonging to seven ligand families) were curated to validate this approach and supplemented with the BDL-Cu-2023 data set as a pool for further ligand optimization. Our workflow retrieves the best possible linear model established from a combination of electronic, steric, and (critically important but frequently overlooked) topological features that were determined using BRR. By coupling BRR with BO, we were able to efficiently screen ligands, even in limited data scenarios, which allowed design principles to be extracted and new ligands to be proposed for the oxy-alkynylation reaction. Overall, the approach presented here enables researchers to optimize ligand selection and design at any stage of experimentation, resulting in more efficient and cost-effective enantioselective reaction development.

## 5 Computational Details

DFT computations of ligands were done at the PBE0-D3(BJ)/def2-SVP level using Gaussian16.^100−86^ For ligands extracted from the literature, 3D coordinates were generated using OpenBabel (version 2.4.1)^88^ and then optimized at the GFN2-xTB level^87^ before final optimization with DFT. The desired structure (chelating groups oriented toward the metal atom) was obtained by adding CuCl_2_ to the molecules before the optimization, as previously reported (see Table S1 for comparison with CuCl geometries).^27,57^ Ligands used in the Ni-catalyzed reactions were optimized with CuCl_2_ and NiF_2_ for comparison (see Table S2). Similar to Cu(I) and Cu(II), the rmsd for the tested structures was found to be lower than 1 Å on average. All electronic features, including NBO analyses,^89^ were performed on the metal-free ligand structures. Atoms for the different NBO charges (atom itself and lone-pair) were defined based on distance to the metal center. The optimized or crystal structure coordinates were used to compute the steric features and build the molecular graphs. For the steric features, both libarvo^90,92^ and Morfeus^91^ were used. Features derived from the molecular graph were generated using the newly developed Moltop Python package. Whenever bond orders are required for a specific feature (such as the Crest flexibility index), these have to be defined explicitly. Supported bond orders currently include ones from NBO analyses, xTB, and RDkit. All Moltop instructions and scripts used in this study are available on GitHub at https://github.com/lcmd-epfl/rafbl and as part of the NaviCat platform at https://github.com/lcmd-epfl/NaviCat. Additionally, the generated data can be explored interactively in the MaterialsCloud repository https://doi.org/10.24435/materialscloud:c0-7z. The Sklearn package^93^ was used for linear models.

## Supporting Information Available

Description and details of data sets; discussion of the ligand geometry optimization; details of ligand featurization methods including complete list of features; and additional model details and related discussion (PDF)
