# Supplementary material for: Reaction-Agnostic Featurization of Bidentate Ligands for Bayesian Ridge Regression of Enantioselectivity
Source: ACS Catal. 2024 Jun 4;14(12):9302–12. doi: 10.1021/acscatal.4c02452 (PMC11197013; doi:10.1021/acscatal.4c02452)
Supplement: Supplementary file 1 — cs4c02452_si_001.pdf [file cs4c02452_si_001.pdf]

Supporting Information for: Reaction-Agnostic Featurization of Bidentate Ligands for Bayesian Ridge Regression of Enantioselectivity

Alexandre A. Schoepfer,[†,‡,¶] Ruben Laplaza,[†,¶] Matthew D. Wodrich,[†,¶] Jerome Waser,[*,‡,¶] and Clémence Corminboeuf[*,†,¶]

†*Laboratory for Computational Molecular Design, Institute of Chemical Sciences and Engineering, École Polytechnique Fédérale de Lausanne (EPFL), 1015 Lausanne, Switzerland*

‡*Laboratory of Catalysis and Organic Synthesis, Institute of Chemical Sciences and Engineering, École Polytechnique Fédérale de Lausanne (EPFL), 1015 Lausanne, Switzerland*

¶*National Center for Competence in Research-Catalysis (NCCR-Catalysis), École Polytechnique Fédérale de Lausanne, 1015 Lausanne, Switzerland*

E-mail: jerome.waser@epfl.ch; clemence.corminboeuf@epfl.ch

S1 Code and data availability

This document contains information regarding the datasets considered in the main text, a benchmark of geometry optimization effects and a complete list defining all features considered in this work. All code and scripts to reproduce all results can be found at `https://github.com/lcmd-epfl/rafbl`, and all the relevant data can be obtained and explored interactively in the MaterialsCloud repository `https://doi.org/10.24435/materialscloud:c0-7z`.

S2 Datasets

All datasets were taken from experimental ligand screening experiments. Scheme S1 displays all ligands considered for the oxy-alkynylation reaction (**OA**).[1] Additional ligands not reported in the original publication were taken from electronic laboratory notebook (ELN) entries. Scheme S2 and S3 display ligands considered for the cyclopropanation (**CP**) and cross-electrophile coupling (**CC**) respectively.[2,3] Finally, Scheme S4 displays ligands used for a Diels-Alder reaction (**DA**).[4–10] For **DA** models, the dimethylamine α-diimine ligand is removed from the training set as its electronic properties are very different from the other ligands.

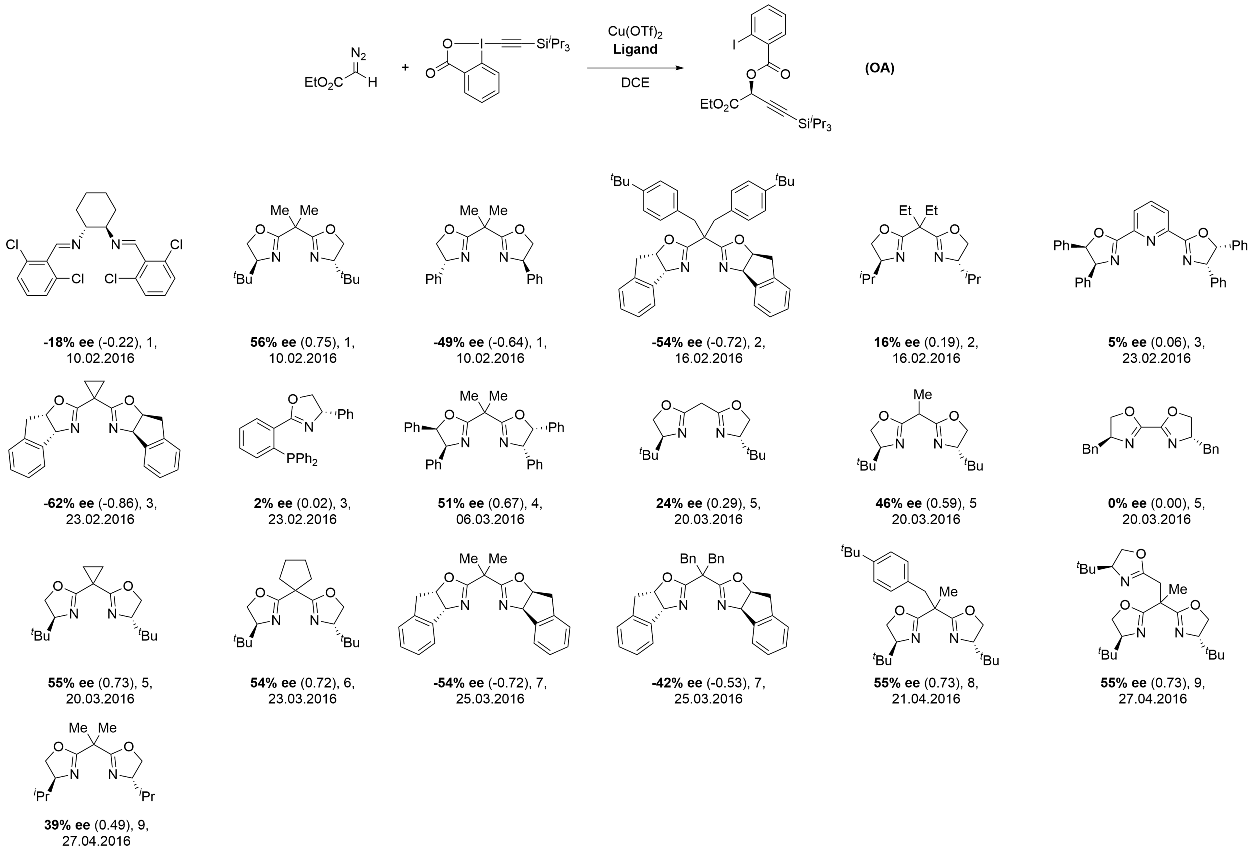

Scheme S1: Ligand screening for the oxy-alkynylation (**OA**) reaction. Enantiomeric excesses (ee), $\Delta\Delta G^{\ddagger}$ in kcal/mol in parentheses, chronological order of the experiment are given below each ligand and the date at which the experiment was started.

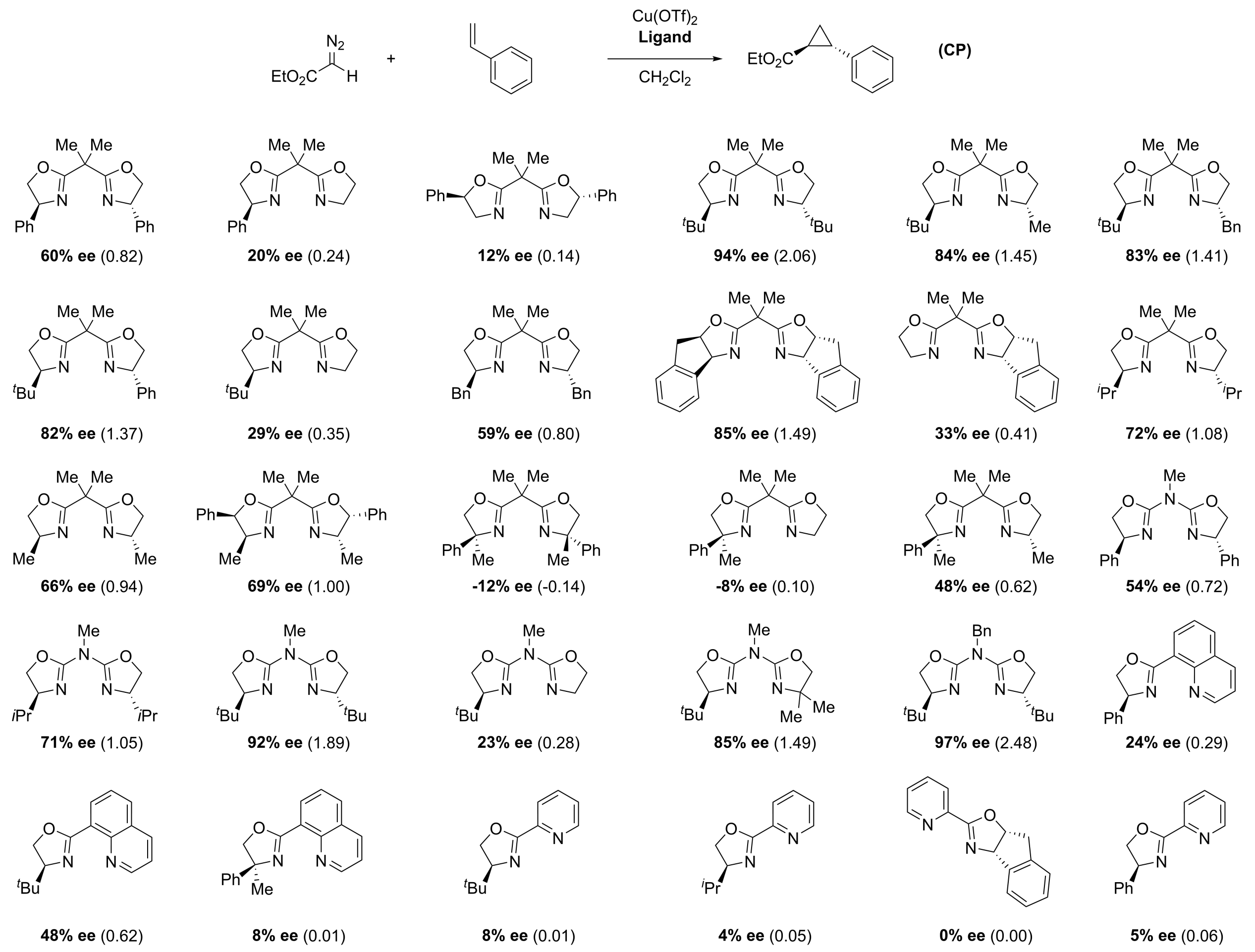

Scheme S2: Ligand screening for the cyclopropanation (**CP**) reaction. Enantiomeric excesses (ee) and $\Delta\Delta G^{\ddagger}$ in kcal/mol in parentheses are given below each ligand.

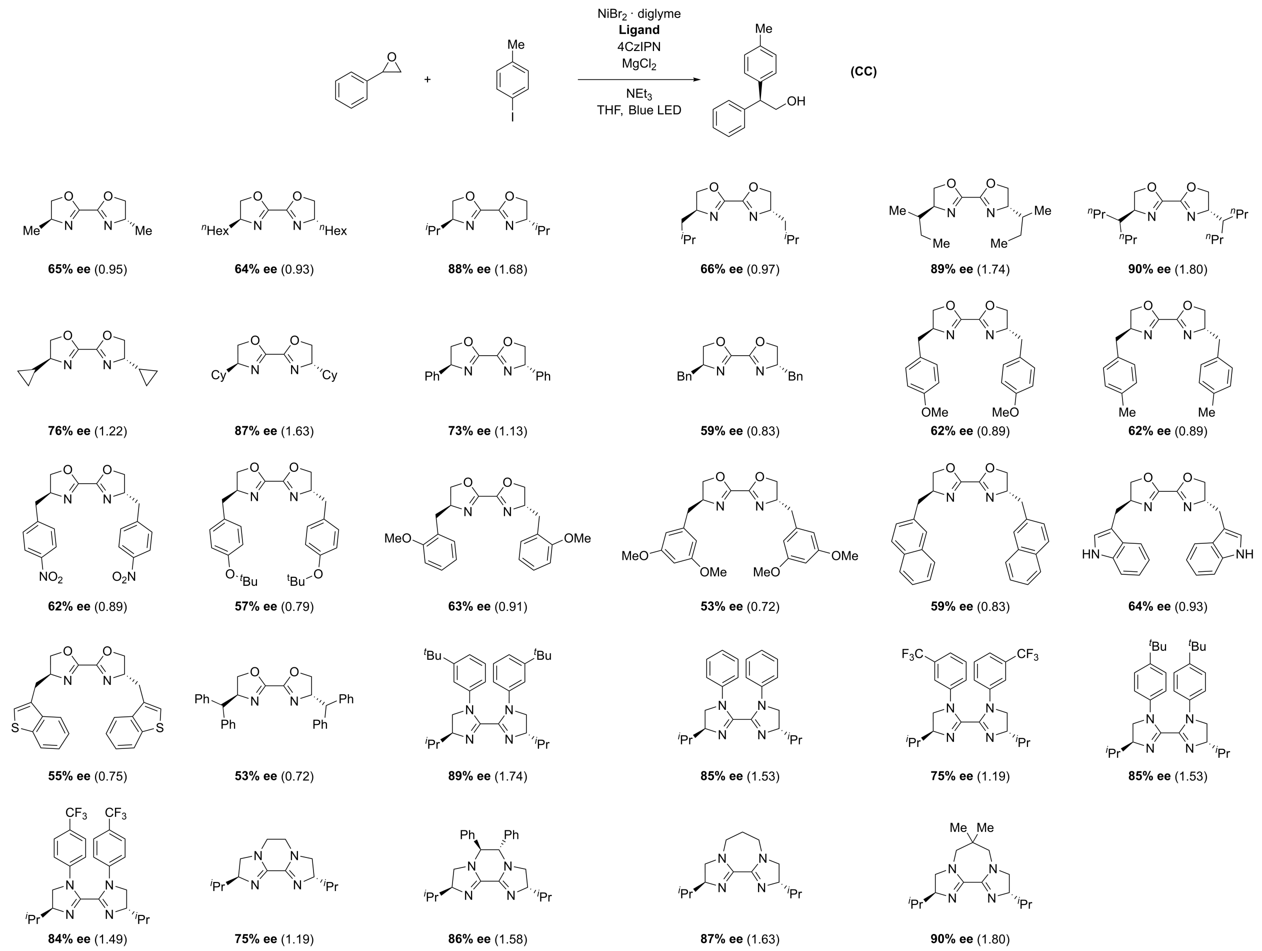

Scheme S3: Ligand screening for the cross-electrophile coupling (**CC**) reaction. Enantiomeric excesses (ee) and $\Delta\Delta G^{\ddagger}$ in kcal/mol in parentheses are given below each ligand.

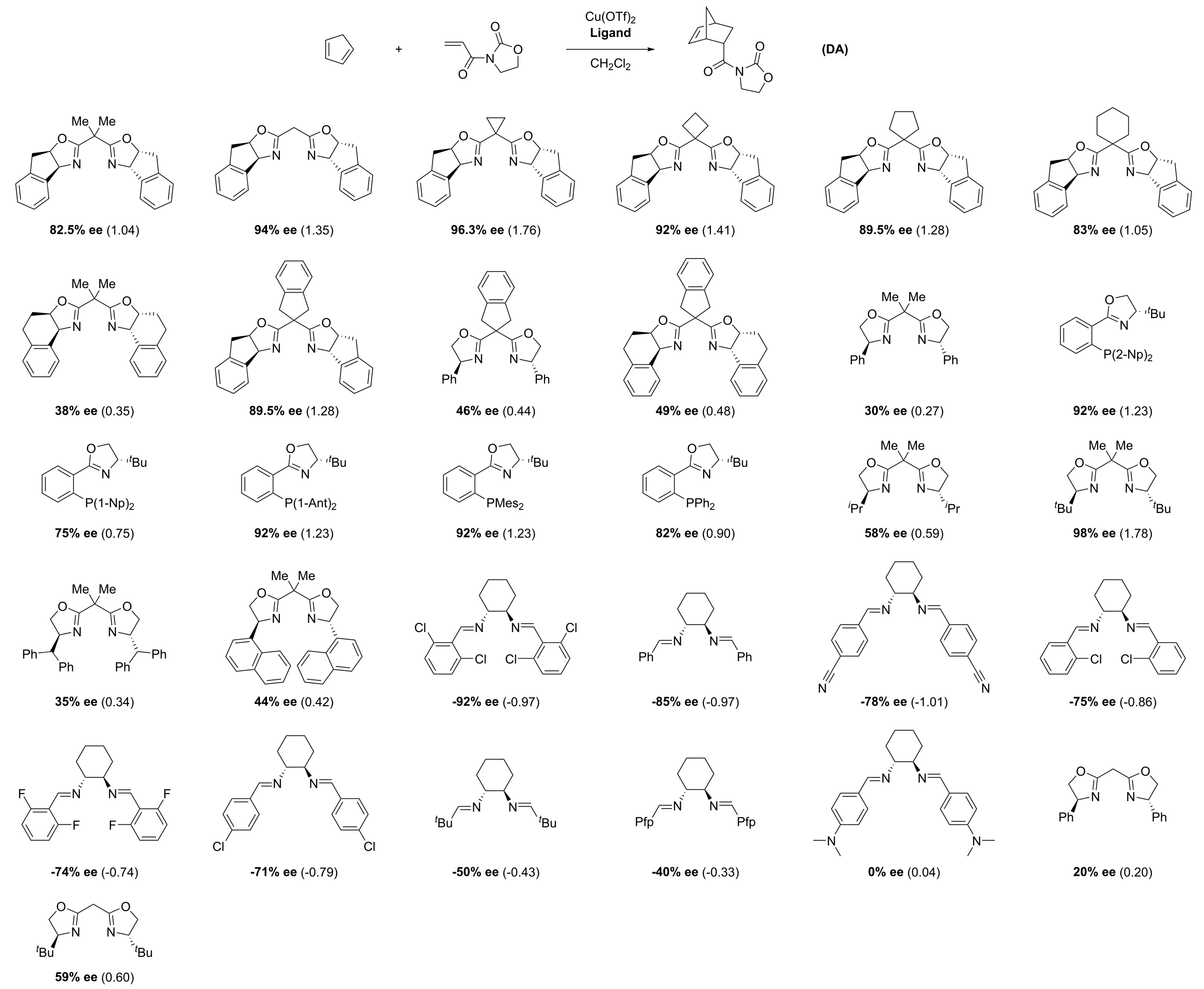

Scheme S4: Ligand screening for the Diels-Alder (**DA**) reaction. Enantiomeric excesses (ee) and $\Delta\Delta G^{\ddagger}$ in kcal/mol in parentheses are given below each ligand.

S2.1 Screening of additional ligands for OA

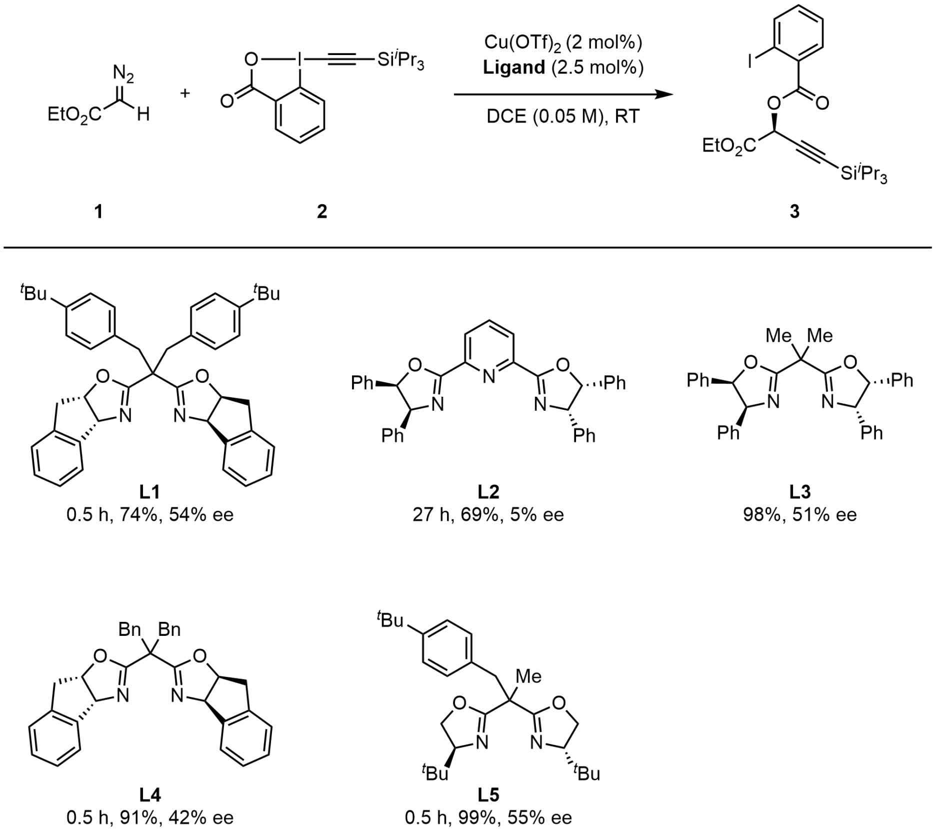

Scheme S5: Additional ligand screening experiments from the ELN. Synthesis of **L1** (opposite enantiomer),[11] **L2**,[12] **L3**,[13] **L4**,[14] and **L5**,[15] are all reported.

To avoid reproducibility issues, experimental procedures of the ELN entries were taken from the original manuscript:[1]

A flame dried 5 mL microwave vial was charged under nitrogen with catalyst (3.00 µmol, 0.02 equiv), ligand (3.75 µmol, 0.025 equiv) and dry DCE (1 mL). The resulting solution was stirred at room temperature for 30 minutes. To this solution was added a mixture of 1-[(triiso-propylsilyl)ethynyl]-1,2-benziodoxol-3(1H)-one (**2**) (0.15 mmol, 1.0 equiv) and ethyl 2-diazoacetate (**1**) (0.30 mmol, 2.0 equiv) in dry DCE (2 mL) in 2 min and the resulting reaction mixture was stirred until the reaction was completed (monitored by TLC, EtOAc:pentane, 1:40 v/v), the solvent was evaporated under reduced pressure and the crude product was purified by column chromatography (EtOAc:pentane, 1:40 v/v) directly without

any further work-up.

S2.2 CSD ligand types

Figure S1 shows the variety of copper ligands found bound to Cu(I) or Cu(II) in the CSD (Cambridge structural database). Besides nitrogen and oxygen, more unusual ligand types with phosphorus and sulfur as binding atoms were also included and successfully featurized with our pipeline.

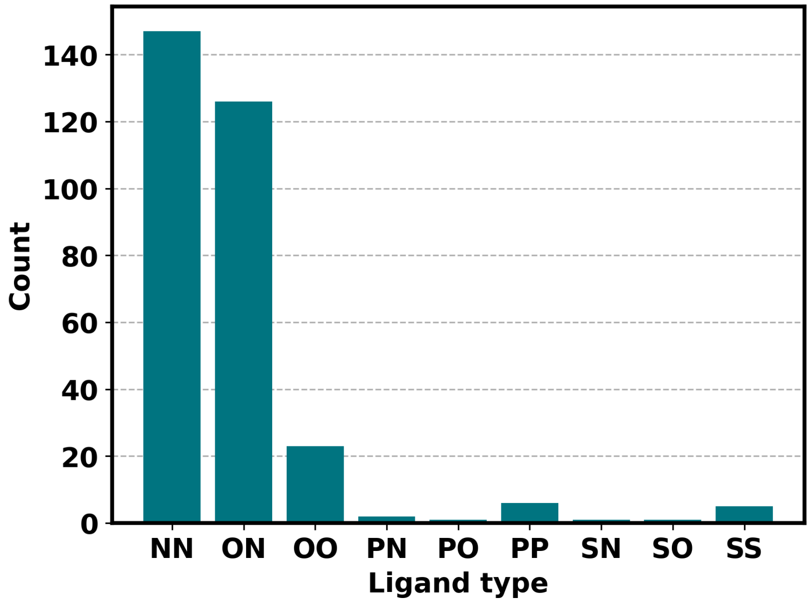

Figure S1: Frequency of the types of bidentate ligand bound to copper extracted from the CSD.

S3 Ligand geometry optimization

To obtain representative geometries for the bidentate ligands under consideration, geometry optimization was performed including a metal atom attached to the binding atoms of the ligands. We benchmarked the importance of the choice of metal and oxidation state by comparing the resulting ligand geometries with Cu(II) vs Cu(I) (Table S1) and Ni(II) (Table S2). In both cases, the mean average root mean squared deviation (RMSD) was deemed low enough, meaning that the overall ligand geometries are not very sensitive to the choice.

Table S1: Root mean square deviation of between Cu(I) and Cu(II) geometries for selected structures.

Filename	RMSD (Å)
001_lig.xyz	1.1307
002_lig.xyz	0.1948
003_lig.xyz	0.6493
004_lig.xyz	0.6872
005_lig.xyz	0.5092
006_lig.xyz	0.8537
007_lig.xyz	0.9462
008_lig.xyz	0.1474
009_lig.xyz	0.3900
010_lig.xyz	0.6102
011_lig.xyz	0.1786
012_lig.xyz	0.1802
013_lig.xyz	1.1138
**Mean**	0.5839

Table S2: Root mean square deviation of between Ni(II) and Cu(II) geometries for selected structures.

Filename	RMSD (Å)
072_lig.xyz	0.1713
074_lig.xyz	0.3689
075_lig.xyz	0.2153
079_lig.xyz	0.4848
080_lig.xyz	0.3739
089_lig.xyz	0.5775
090_lig.xyz	1.0696
091_lig.xyz	0.3597
092_lig.xyz	0.3623
093_lig.xyz	0.3364
094_lig.xyz	0.3781
095_lig.xyz	0.3012
096_lig.xyz	0.3656
097_lig.xyz	0.4307
098_lig.xyz	1.7075
099_lig.xyz	1.905
100_lig.xyz	0.2272
**Mean**	0.5668

S4 Featurization of bidentate ligands

S4.1 General procedure

To align ligands in space, we define the metal center as the origin of the coordinates. Both chelating groups are placed in the xz-plane and rotated so that the dot product of their coordinates follows the negative z-axis. The center of mass is then finally defined to have a positive x-value. This is an important step for asymmetric ligands, ensuring a consistent alignment. This alignment allows the molecular volume of the ligand to be split into eight octants in a reproducible fashion. The only prerequisite the molecule has to possess for this alignment is two atoms binding to a metal center.

Molecular graphs are constructed from 3D coordinates using covalent radii to assign bonds. The resulting graph is then used to calculate topological features, of which many had been developed for medicinal chemistry.[16,17] We split the features obtained from *Moltop*, the Python package developed for this work, into three subclasses: bond fragment-based, ones that require bond orders, and purely graph theoretical features. The first class includes indices developed by Kier and Hall,[16,18–23] which are derived from the number of unique n bond fragments. Either covalent radii, or valences and atom hyperconjugations are used to differentiate between heteroatoms. Additionally, we implemented a new variation of the Kier $^n\kappa$ indices based on bond length obtained directly from the molecular geometries, which we refer to as $^n\kappa_\beta$. The second class of indices requires bond orders and include local and global simple (LS and GS) indices, and the flexibility index implemented in the conformational ensemble generation tool Crest.[24,25] The last family of topological features only requires connectivity information and includes the graph-theoretical Wiener, Hosoya Z, and Balaban J indices,[26–28] which are related to the global connectedness and compactness of the molecule. Rigidity/flexibility can also be accounted by considering ensembles of conformers,[29,30] but such indices are more computationally expensive than the ones proposed in this work.

Features are classified in three main classes, electronic, steric and topological. Steric and

topological features can be intensive or extensive. Extensive properties have the advantage of describing the general size of the molecule. As such, indirectly, they encode the number of atoms and the general complexity and constitution of the ligand.

S4.2 Ligand space

The proposed featurization strategy yields 232 features consistently for any possible bidentate ligand, and concatenating these features provides a general representation that covers the chemical space of bidentate ligands. Using dimensionality reduction, in Figure S2 we visualize the space of all 100 experimental ligands (see Section 2.1 of the main text) that belong to seven well-defined classes. An ideal set of features should be able to cluster together ligands belonging to the same class, as they would have many similar values, while also keeping clusters of similar classes closer and very different ones far away.

Figure S2 shows dimensionality reduction plots of five different representations that are built by concatenating subsets of features originating from our featurization strategy (see Section S4.1). Noticeably, the distribution of points is highly dependent on the subset of features used. Figure S2A shows the PCA (principal component analysis) plot obtained using the complete representation with all 232 available features, while S2B only includes electronic and steric features. Panels S2C, S2D, and S2E were obtained from the three main feature classes each: electronics, sterics, and topological.

In general, S2A, where all features are combined, yields as expected the best compromise. α-diimines (dark blue) do not overlap with the IndaBOX class, similar to the purely topological map S2E. Steric and topological information is also retained for PyOX (red) ligands, similarly to S2D and S2E. PhosOX ligands (light blue) are electronically very different from the other classes which is apparent in S2C. Both S2A and S2B put this class on the border. The remaining ligand classes 1,2-BOX, 1,3-BOX, and 1,2-B2IM are difficult to discern from each other. Arguably, for 1,2-B2IM, steric features can differentiate them from the other ligand classes as seen in S2D. This observation becomes less obvious in S2A. Finally, 1,2-BOX, and

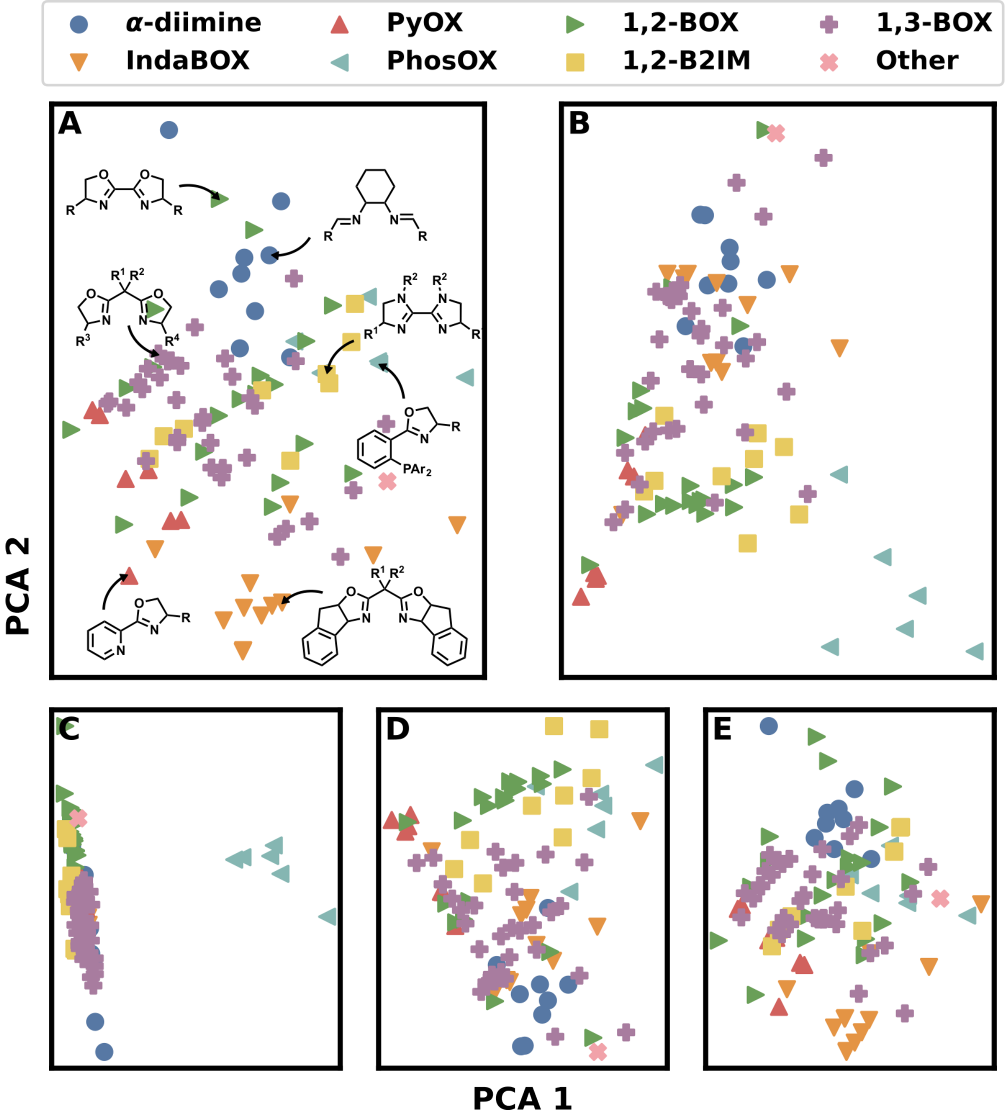

Figure S2: PCA (principal component analysis) map of the feature space for all ligands. (A) All available features. (B) Electronic and steric features. (C) Only electronics. (D) Only sterics. (E) Topology, geometry, and constitution.

1,3-BOX mostly differ by their electronic properties (S2C).

In conclusion, we show that the proposed featurization strategy is able to uniquely represent bidentate ligands while preserving chemically intuitive similarity and dissimilarity between different ligand classes. Topology is shown to help to better discriminate between certain ligand classes and subclasses, especially IndaBOX ligands, in which steric and electronic features are not able to capture the differences with respect to similar ligand classes.

S4.3 Full feature list

Below, we provide an exhaustive list of the 232 features considered in this work for bidentate ligands. Note that all topological descriptors can be generated using the *Moltop* package and associated scripts available in `https://github.com/lcmd-epfl/rafbl`.

0. **Handle**; **Symbol**; **Unit**; **Name**; **Class**; **Description**; **Reference**

1. *lumo*; ϵ_{LUMO}; Hartree; LUMO energy; electronic; Energy of the Lowest unoccupied molecular orbital; -

2. *homo*; ϵ_{HOMO}; Hartree; HOMO energy; electronic; Energy of the Highest occupied molecular orbital; -

3. *gap*; $\Delta\epsilon$; Hartree; LUMO-HOMO gap; electronic; Energy difference between LUMO and HOMO; -

4. *dipole*; μ; Debye; Dipole moment; electronic; Dipole moment of molecule; -

5. *nbo1*; NBO_1; Hartree; NBO energy 1; electronic; NBO energy of the first coordinating atom; -

6. *nbo2*; NBO_2; Hartree; NBO energy 2; electronic; NBO energy of the second coordinating atom; -

7. *nbo-*; ΔNBO; Hartree; NBO energy difference; electronic; NBO energy difference of both coordinating atoms; -

8. *nbo+*; ΣNBO; Hartree; NBO energy sum; electronic; NBO energy sum of both coordinating atoms; -

9. *nbo**; ΠNBO; Hartree; NBO energy product; electronic; NBO energy product of both coordinating atoms; -

10. *nbom*; μNBO; Hartree; Mean NBO energy; electronic; Mean NBO energy of both coordinating atoms; -

11. *nbo1/2*; NBO_1/NBO_2; Hartree; NBO energy ratio; electronic; Ration between NBO energy 1 and NBO energy 2; -

12. *cnbo1*; q_1; Coulomb; Natural charge 1; electronic; Natural charge of the first coordinating atom; -

13. *cnbo2*; q_2; Coulomb; Natural charge 2; electronic; Natural charge of the second coordinating atom; -

14. *cnbo-*; Δq; Coulomb; Natural charge difference; electronic; Natural charge difference of both coordinating atoms; -

15. *cnbo+*; Σq; Coulomb; Natural charge sum; electronic; Natural charge sum of both coordinating atoms; -

16. *cnbo**; Πq; Coulomb; Natural charge product; electronic; Natural charge product of both coordinating atoms; -

17. *cnbom*; μq; Coulomb; Mean Natural charge; electronic; Mean natural charge of both coordinating atoms; -

18. *cnbo1/2*; q_1/q_2; Coulomb; Natural charge ratio; electronic; Ration between Natural charge 1 and Natural charge 2; -

19. *onbo1*; e_1; -; Natural occupancy 1; electronic; Natural occupancy of the first coordinating atom; -

20. *onbo2*; e_2; -; Natural occupancy 2; electronic; Natural occupancy of the second coordinating atom; -

21. *onbo-*; Δe; -; Natural occupancy difference; electronic; Natural occupancy difference of both coordinating atoms; -

22. *onbo+*; Σe; -; Natural occupancy sum; electronic; Natural occupancy sum of both coordinating atoms; -

23. *onbo**; Πe; -; Natural occupancy product; electronic; Natural occupancy product of both coordinating atoms; -

24. *onbom*; μe; -; Mean Natural occupancy; electronic; Mean natural occupancy of both coordinating atoms; -

25. *onbo1/2*; e_1/e_2; -; Natural occupancy ratio; electronic; Ration between Natural occupancy 1 and Natural occupancy 2; -

26. *na*; n_a; -; Atom number; topological extensive; Number if atoms in the molecule; -

27. *na_an*; n_{anh}; -; Non-hydrogen atom number; topological extensive; Number of non-hydrogen atoms; -

28. *nb*; n_b; -; Bond number; topological extensive; Number of bonds; -

29. *estrada*; EE; -; Estrada index; topological extensive; Folding degree of molecule ; `https://doi.org/10.1016/S0009-2614(00)00158-5`

30. *wiener*; W; -; Wiener index; topological extensive; Index describing molecular branching ; https://doi.org/10.1021/ja01193a005

31. *global_eff*; E_{glob}; -; Global efficiency; topological extensive; Measure of how efficient information in a graph is exchanged ; https://doi.org/10.1103/PhysRevLett.87.198701

32. *balaban*; J; -; Balaban J index; topological extensive; Index describing molecular branching for similar molecules; https://doi.org/10.1016/0009-2614(82)80009-2

33. *hosoya*; Z; -; Hosoya Z index; topological extensive; Number of possible matching in a graph; https://doi.org/10.1246/bcsj.44.2332

34. *zagreb1*; M_1; -; Zagreb 1 index; topological extensive; Atom-wise connectivity; https://doi.org/10.1016/0009-2614(72)85099-1

35. *zagreb2*; M_2; -; Zagreb 2 index; topological extensive; Atom-pairwise connectivity; https://doi.org/10.1016/0009-2614(72)85099-1

36. *global_simplex*; GS; -; Global Simplex; topological extensive; Flexibility index including bond-types, rings, and branching; https://doi.org/10.1016/0898-5529(90)90163-3

37. *kier_a*; α; -; Kier alpha; topological extensive; Kier heteroatom count based on covalent radii; -

38. *kier_b*; β; -; Kier beta; topological extensive; Kier heteroatom count based on bond lengths; -

39. *kier_al*; αl; -; Legacy Kier alpha; topological extensive; Legacy Kier heteroatom count based on covalent radii; https://doi.org/10.1002/qsar.19860050103

40. *0k*; ${}^0\kappa$; -; 0-order Kier index; topological extensive; 0-bond-fragment atom diversity index; https://doi.org/10.1002/qsar.19870060103

41. *1k*; $^{1}\kappa$; -; 1-order Kier index; topological extensive; 1-bond-fragment shape index; https://doi.org/10.1002/qsar.19860050102

42. *2k*; $^{2}\kappa$; -; 2-order Kier index; topological extensive; 2-bond-fragment shape index; https://doi.org/10.1002/qsar.19850040303

43. *3k*; $^{3}\kappa$; -; 3-order Kier index; topological extensive; 3-bond-fragment shape index; https://doi.org/10.1002/qsar.19860050102

44. *1ka*; $^{1}\kappa_{\alpha}$; -; 1-order atom Kier index; topological extensive; 1-bond-fragment atom shape index; -

45. *2ka*; $^{2}\kappa_{\alpha}$; -; 2-order atom Kier index; topological extensive; 2-bond-fragment atom shape index; -

46. *3ka*; $^{3}\kappa_{\alpha}$; -; 3-order atom Kier index; topological extensive; 3-bond-fragment atom shape index; -

47. *1kb*; $^{1}\kappa_{\beta}$; -; 1-order bond Kier index; topological extensive; 1-bond-fragment bond shape index; -

48. *2kb*; $^{2}\kappa_{\beta}$; -; 2-order bond Kier index; topological extensive; 2-bond-fragment bond shape index; -

49. *3kb*; $^{3}\kappa_{\beta}$; -; 3-order bond Kier index; topological extensive; 3-bond-fragment bond shape index; -

50. *1kal*; $^{1}\kappa_{\alpha l}$; -; 1-order legacy atom Kier index; topological extensive; 1-bond-fragment legacy atom shape index; https://doi.org/10.1002/qsar.19860050103

51. *2kal*; $^{2}\kappa_{\alpha l}$; -; 2-order legacy atom Kier index; topological extensive; 2-bond-fragment legacy atom shape index; https://doi.org/10.1002/qsar.19860050103

52. *3kal*; ${}^{3}\kappa_{\alpha l}$; -; 3-order legacy atom Kier index; topological extensive; 3-bond-fragment legacy atom shape index; `https://doi.org/10.1002/qsar.19860050103`

53. *k_xia*; $\Xi\alpha$; -; Atom steric effect index; topological extensive; atom and κ_α based steric effect index ; -

54. *k_xib*; $\Xi\beta$; -; Bond steric effect index; topological extensive; bond and κ_β based steric effect index ; -

55. *k_xial*; $\Xi\alpha l$; -; Legacy atom steric effect index; topological extensive; Legacy atom and $\kappa_{\alpha l}$ based steric effect index ; `https://doi.org/10.1002/qsar.19870060305`

56. *redu*; *R*; -; Redundancy; topological extensive; Measure of atom type variety ; Brillouin & Leon, *Science and information theory*, (**1962**).

57. *0chi*; ${}^{0}\chi$; -; 0-order connectivity index; topological extensive; 0-order χ connectivity; `https://doi.org/10.1002/qsar.19890080406`

58. *1chi*; ${}^{1}\chi$; -; 1-order connectivity index; topological extensive; 1-order χ connectivity; `https://doi.org/10.1002/qsar.19890080406`

59. *2chi*; ${}^{2}\chi$; -; 2-order connectivity index; topological extensive; 2-order χ connectivity; `https://doi.org/10.1002/qsar.19890080406`

60. *3chi*; ${}^{3}\chi$; -; 3-order connectivity index; topological extensive; 3-order χ connectivity; `https://doi.org/10.1002/qsar.19890080406`

61. *4chi*; ${}^{4}\chi$; -; 4-order connectivity index; topological extensive; 4-order χ connectivity; `https://doi.org/10.1002/qsar.19890080406`

62. *5chi*; ${}^{5}\chi$; -; 5-order connectivity index; topological extensive; 5-order χ connectivity; `https://doi.org/10.1002/qsar.19890080406`

63. *0chiv*; ${}^0\chi^v$; -; 0-order connectivity index with valence; topological extensive; 0-order χ connectivity with valence; `https://doi.org/10.1002/qsar.19890080406`

64. *1chiv*; ${}^1\chi^v$; -; 1-order connectivity index with valence; topological extensive; 1-order χ connectivity with valence; `https://doi.org/10.1002/qsar.19890080406`

65. *2chiv*; ${}^2\chi^v$; -; 2-order connectivity index with valence; topological extensive; 2-order χ connectivity with valence; `https://doi.org/10.1002/qsar.19890080406`

66. *3chiv*; ${}^3\chi^v$; -; 3-order connectivity index with valence; topological extensive; 3-order χ connectivity with valence; `https://doi.org/10.1002/qsar.19890080406`

67. *4chiv*; ${}^4\chi^v$; -; 4-order connectivity index with valence; topological extensive; 4-order χ connectivity with valence; `https://doi.org/10.1002/qsar.19890080406`

68. *5chiv*; ${}^5\chi^v$; -; 5-order connectivity index with valence; topological extensive; 5-order χ connectivity with valence; `https://doi.org/10.1002/qsar.19890080406`

69. *T*; τ; -; Global topological state index; topological extensive; Measure of topological equivalence in whole molecule; `https://doi.org/10.1002/qsar.19900090207`

70. *Tnbo1*; S_1^v; -; Local topological state index 1; topological extensive; Topological state for the first coordinating atom; `https://doi.org/10.1002/qsar.19900090207`

71. *Tnbo2*; S_2^v; -; Local topological state index 2; topological extensive; Topological state for the second coordinating atom; `https://doi.org/10.1002/qsar.19900090207`

72. *s0chi*; $S^0\chi$; -; Sum of 0-order χ indices; topological extensive; -; `https://doi.org/10.1002/qsar.19890080406`

73. *s1chi*; $S^1\chi$; -; Sum of 1-order χ indices; topological extensive; -; `https://doi.org/10.1002/qsar.19890080406`

74. *s2chi*; $S^2\chi$; -; Sum of 2-order χ indices; topological extensive; -; https://doi.org/10.1002/qsar.19890080406

75. *s3chi*; $S^3\chi$; -; Sum of 3-order χ indices; topological extensive; -; https://doi.org/10.1002/qsar.19890080406

76. *s4chi*; $S^4\chi$; -; Sum of 4-order χ indices; topological extensive; -; https://doi.org/10.1002/qsar.19890080406

77. *s5chi*; $S^5\chi$; -; Sum of 5-order χ indices; topological extensive; -; https://doi.org/10.1002/qsar.19890080406

78. *bpa*; n_b/n_a; -; Bonds per atoms; topological intensive; Number of bonds per atoms; -

79. *crest_flex*; μ_f; -; Crest flexibility index; topological intensive; Flexibility index used in crest for to estimate length of meta-physiscs; https://doi.org/10.1039/C9CP06869D

80. *angle*; Θ; Radian; Bite angle; topological intensive; Angle of the metal center and the two coordinating atoms; -

81. *t_ova*; O; -; Ovality; topological intensive; Deviation from a perfect sphere; -

82. *t_ova_nh*; O_{nh}; -; Hydrogen-free ovality; topological intensive; Hydrogen-free deviation from a perfect sphere; -

83. *festrada*; $\hat{EE}$; -; Relative Estrada index; topological intensive; Estrada index divided by number of heavy atoms; -

84. *fwiener*; $\hat{W}$; -; Relative Wiener index; topological intensive; Wiener index divided by number of heavy atoms; -

85. *fglobal_eff*; $\hat{E_{glob}}$; -; Relative global efficiency; topological intensive; Global efficiency divided by number of heavy atoms; -

86. *fbalaban*; $\hat{J}$; -; Relative Balaban J index; topological intensive; Balaban J index divided by number of heavy atoms; -

87. *fhosoya*; $\hat{Z}$; -; Relative Hosoya Z index; topological intensive; Hosoya Z index divided by number of heavy atoms; -

88. *fzagreb1*; $\hat{M}_1$; -; Relative Zagreb 1 index; topological intensive; Zagreb 1 index divided by number of heavy atoms; -

89. *fzagreb2*; $\hat{M}_2$; -; Relative Zagreb 2 index; topological intensive; Zagreb 2 index divided by number of heavy atoms; -

90. *fkier_a*; $\hat{\alpha}$; -; Relative Kier alpha; topological intensive; α divided by number of heavy atoms; -

91. *fkier_b*; $\hat{\beta}$; -; Relative Kier beta; topological intensive; β divided by number of heavy atoms; -

92. *fkier_al*; $\hat{\alpha l}$; -; Relative legacy Kier alpha; topological intensive; αl divided by number of heavy atoms; -

93. *f0k*; ${}^0\hat{\kappa}$; -; Relative ${}^0\kappa$; topological intensive; ${}^0\kappa$ divided by number of heavy atoms; -

94. *f1k*; ${}^1\hat{\kappa}$; -; Relative ${}^1\kappa$; topological intensive; ${}^1\kappa$ divided by number of heavy atoms; -

95. *f2k*; ${}^2\hat{\kappa}$; -; Relative ${}^2\kappa$; topological intensive; ${}^2\kappa$ divided by number of heavy atoms; -

96. *f3k*; ${}^3\hat{\kappa}$; -; Relative ${}^3\kappa$; topological intensive; ${}^3\kappa$ divided by number of heavy atoms; -

97. *f1ka*; ${}^1\hat{\kappa}_\alpha$; -; Relative ${}^1\kappa_\alpha$; topological intensive; ${}^1\kappa_\alpha$ divided by number of heavy atoms; -

98. *f2ka*; ${}^2\hat{\kappa}_\alpha$; -; Relative ${}^2\kappa_\alpha$; topological intensive; ${}^2\kappa_\alpha$ divided by number of heavy atoms; -

99. *f3ka*; $^3\hat{\kappa}_\alpha$; -; Relative $^3\kappa_\alpha$; topological intensive; $^3\kappa_\alpha$ divided by number of heavy atoms; -

100. *f1kb*; $^1\hat{\kappa}_\beta$; -; Relative $^1\kappa_\beta$; topological intensive; $^1\kappa_\beta$ divided by number of heavy atoms; -

101. *f2kb*; $^2\hat{\kappa}_\beta$; -; Relative $^2\kappa_\beta$; topological intensive; $^2\kappa_\beta$ divided by number of heavy atoms; -

102. *f3kb*; $^3\hat{\kappa}_\beta$; -; Relative $^3\kappa_\beta$; topological intensive; $^3\kappa_\beta$ divided by number of heavy atoms; -

103. *f1kal*; $^1\hat{\kappa}_{\alpha l}$; -; Relative $^1\hat{\kappa}_{\alpha l}$; topological intensive; $^1\hat{\kappa}_{\alpha l}$ divided by number of heavy atoms; -

104. *f2kal*; $^2\hat{\kappa}_{\alpha l}$; -; Relative $^2\hat{\kappa}_{\alpha l}$; topological intensive; $^2\hat{\kappa}_{\alpha l}$ divided by number of heavy atoms; -

105. *f3kal*; $^3\hat{\kappa}_{\alpha l}$; -; Relative $^3\hat{\kappa}_{\alpha l}$; topological intensive; $^3\hat{\kappa}_{\alpha l}$ divided by number of heavy atoms; -

106. *k_phi*; Φ; -; Kier flexibility; topological intensive; Flexibility of molecules based on $^1\kappa$, $^2\kappa$ and number of heavy atoms; -

107. *k_phia*; $\Phi\alpha$; -; Atom Kier flexibility; topological intensive; Flexibility of molecules based on $^1\kappa_\alpha$, $^2\kappa_\alpha$ and number of heavy atoms; -

108. *k_phib*; $\Phi\beta$; -; Bond Kier flexibility; topological intensive; Flexibility of molecules based on $^1\kappa_\beta$, $^2\kappa_\beta$ and number of heavy atoms; -

109. *k_phial*; $\Phi\alpha l$; -; Legacy atom Kier flexibility; topological intensive; Flexibility of molecules based on $^1\kappa_{\alpha l}$, $^2\kappa_{\alpha l}$ and number of heavy atoms; `https://doi.org/10.1002/qsar.19890080307`

110. *k_ups*; Υ; -; Extended Kier flexibility; topological intensive; Flexibility of molecules based on ${}^1\kappa$, ${}^2\kappa$, ${}^3\kappa$, and number of heavy atoms; -

111. *k_upsa*; Υ_α; -; Extended atom Kier flexibility; topological intensive; Flexibility of molecules based on ${}^1\kappa_\alpha$, ${}^2\kappa_\alpha$, ${}^3\kappa_\alpha$, and number of heavy atoms; -

112. *k_upsb*; Υ_β; -; Extended bond Kier flexibility; topological intensive; Flexibility of molecules based on ${}^1\kappa_\beta$, ${}^2\kappa_\beta$, ${}^3\kappa_\beta$, and number of heavy atoms; -

113. *k_upsal*; $\Upsilon_{\alpha l}$; -; Extended legacy atom Kier flexibility; topological intensive; Flexibility of molecules based on ${}^1\kappa_{\alpha l}$, ${}^2\kappa_{\alpha l}$, ${}^3\kappa_{\alpha l}$, and number of heavy atoms; -

114. *f0chi*; ${}^0\hat{\chi}$; ; Relative ${}^0\chi$ index; topological intensive; ${}^0\chi$ divided by number of heavy atoms; -

115. *f1chi*; ${}^1\hat{\chi}$; ; Relative ${}^1\chi$ index; topological intensive; ${}^1\chi$ divided by number of heavy atoms; -

116. *f2chi*; ${}^2\hat{\chi}$; ; Relative ${}^2\chi$ index; topological intensive; ${}^2\chi$ divided by number of heavy atoms; -

117. *f3chi*; ${}^3\hat{\chi}$; ; Relative ${}^3\chi$ index; topological intensive; ${}^3\chi$ divided by number of heavy atoms; -

118. *f4chi*; ${}^4\hat{\chi}$; ; Relative ${}^4\chi$ index; topological intensive; ${}^4\chi$ divided by number of heavy atoms; -

119. *f5chi*; ${}^5\hat{\chi}$; ; Relative ${}^5\chi$ index; topological intensive; ${}^5\chi$ divided by number of heavy atoms; -

120. *f0chiv*; ${}^0\hat{\chi}^v$; ; Relative ${}^0\chi^v$ index; topological intensive; ${}^0\chi^v$ divided by number of heavy atoms; -

121. *f1chiv*; ${}^{1}\hat{\chi}^{v}$; ; Relative ${}^{1}\chi^{v}$ index; topological intensive; ${}^{1}\chi^{v}$ divided by number of heavy atoms; -

122. *f2chiv*; ${}^{2}\hat{\chi}^{v}$; ; Relative ${}^{2}\chi^{v}$ index; topological intensive; ${}^{2}\chi^{v}$ divided by number of heavy atoms; -

123. *f3chiv*; ${}^{3}\hat{\chi}^{v}$; ; Relative ${}^{3}\chi^{v}$ index; topological intensive; ${}^{3}\chi^{v}$ divided by number of heavy atoms; -

124. *f4chiv*; ${}^{4}\hat{\chi}^{v}$; ; Relative ${}^{4}\chi^{v}$ index; topological intensive; ${}^{4}\chi^{v}$ divided by number of heavy atoms; -

125. *f5chiv*; ${}^{5}\hat{\chi}^{v}$; ; Relative ${}^{5}\chi^{v}$ index; topological intensive; ${}^{5}\chi^{v}$ divided by number of heavy atoms; -

126. *fT*; -; ; Relative topological state index; topological intensive; τ divided by number of heavy atoms; -

127. *d0chi*; $D^{0}\chi$; ; Difference of 0-order χ indices; topological intensive; -; -

128. *d1chi*; $D^{1}\chi$; ; Difference of 1-order χ indices; topological intensive; -; -

129. *d2chi*; $D^{2}\chi$; ; Difference of 2-order χ indices; topological intensive; -; -

130. *d3chi*; $D^{3}\chi$; ; Difference of 3-order χ indices; topological intensive; -; -

131. *d4chi*; $D^{4}\chi$; ; Difference of 4-order χ indices; topological intensive; -; -

132. *d5chi*; $D^{5}\chi$; ; Difference of 5-order χ indices; topological intensive; -; -

133. *Tenbo1*; I_1; -; Electro-topological state index 1; topological intensive; Topological state index including electronic properties of coordinating atom 1; `https://doi.org/10.1002/qsar.19910100108`

134. *Tenbo2*; I_2; -; Electro-topological state index 2; topological intensive; Topological state index including electronic properties of coordinating atom 2; https://doi.org/10.1002/qsar.19910100108

135. *t_vol*; V; Å^3; Total volume; steric extensive; Total volume including hydrogens; -

136. *t_sur*; S; Å^2; Total surface; steric extensive; Total surface including hydrogens; -

137. *o1*; O^1; Å^3; $O(x, y, z)$ octant; steric extensive; -; -

138. *o2*; O^2; Å^3; $O(-x, y, z)$ octant; steric extensive; -; -

139. *o3*; O^3; Å^3; $O(-x, -y, z)$ octant; steric extensive; -; -

140. *o4*; O^4; Å^3; $O(x, -y, z)$ octant; steric extensive; -; -

141. *o5*; O^5; Å^3; $O(x, -y, -z)$ octant; steric extensive; -; -

142. *o6*; O^6; Å^3; $O(-x, -y, -z)$ octant; steric extensive; -; -

143. *o7*; O^7; Å^3; $O(-x, y, -z)$ octant; steric extensive; -; -

144. *o8*; O^8; Å^3; $O(x, y, -z)$ octant; steric extensive; -; -

145. *oq1*; Q^1; Å^3; $Q(x, y)$ quadrant; steric extensive; -; -

146. *oq2*; Q^2; Å^3; $Q(-x, y)$ quadrant; steric extensive; -; -

147. *oq3*; Q^3; Å^3; $Q(-x, -y)$ quadrant; steric extensive; -; -

148. *oq4*; Q^4; Å^3; $Q(x, -y)$ quadrant; steric extensive; -; -

149. *oh1*; H^1; Å^3; $H(x)$ half; steric extensive; -; -

150. *oh2*; H^2; Å^3; $H(y)$ half; steric extensive; -; -

151. *oh3*; H^3; Å^3; $H(z)$ half; steric extensive; -; -

152. *oh4*; H^4; $\mathring{A}^3$; $H(-x)$ half; steric extensive; -; -

153. *oh5*; H^5; $\mathring{A}^3$; $H(-y)$ half; steric extensive; -; -

154. *oh6*; H^6; $\mathring{A}^3$; $H(-z)$ half; steric extensive; -; -

155. *o_t*; V^o; $\mathring{A}^3$; Octant volumes; steric extensive; -; -

156. *t_vol_nh*; V_{nH}; $\mathring{A}^3$; H-free total volume; steric extensive; -; -

157. *t_sur_nh*; S_{nH}; $\mathring{A}^2$; H-free total surface; steric extensive; -; -

158. *onh1*; O^1_{nH}; $\mathring{A}^3$; H-free $O(x, y, z)$ octant; steric extensive; -; -

159. *onh2*; O^2_{nH}; $\mathring{A}^3$; H-free $O(-x, y, z)$ octant; steric extensive; -; -

160. *onh3*; O^3_{nH}; $\mathring{A}^3$; H-free $O(-x, -y, z)$ octant; steric extensive; -; -

161. *onh4*; O^4_{nH}; $\mathring{A}^3$; H-free $O(x, -y, z)$ octant; steric extensive; -; -

162. *onh5*; O^5_{nH}; $\mathring{A}^3$; H-free $O(x, -y, -z)$ octant; steric extensive; -; -

163. *onh6*; O^6_{nH}; $\mathring{A}^3$; H-free $O(-x, -y, -z)$ octant; steric extensive; -; -

164. *onh7*; O^7_{nH}; $\mathring{A}^3$; H-free $O(-x, y, -z)$ octant; steric extensive; -; -

165. *onh8*; O^8_{nH}; $\mathring{A}^3$; H-free $O(x, y, -z)$ octant; steric extensive; -; -

166. *onhq1*; Q^1_{nH}; $\mathring{A}^3$; H-free $Q(x, y)$ quadrant; steric extensive; -; -

167. *onhq2*; Q^2_{nH}; $\mathring{A}^3$; H-free $Q(-x, y)$ quadrant; steric extensive; -; -

168. *onhq3*; Q^3_{nH}; $\mathring{A}^3$; H-free $Q(-x, -y)$ quadrant; steric extensive; -; -

169. *onhq4*; Q^4_{nH}; $\mathring{A}^3$; H-free $Q(x, -y)$ quadrant; steric extensive; -; -

170. *onhh1*; H^1_{nH}; $\mathring{A}^3$; H-free $H(x)$ half; steric extensive; -; -

171. *onhh2*; H^2_{nH}; $\mathring{A}^3$; H-free $H(y)$ half; steric extensive; -; -

172. *onhh3*; H^3_{nH}; $\mathring{A}^3$; H-free $H(z)$ half; steric extensive; -; -

173. *onhh4*; H^4_{nH}; $\mathring{A}^3$; H-free $H(-x)$ half; steric extensive; -; -

174. *onhh5*; H^5_{nH}; $\mathring{A}^3$; H-free $H(-y)$ half; steric extensive; -; -

175. *onhh6*; H^6_{nH}; $\mathring{A}^3$; H-free $H(-z)$ half; steric extensive; -; -

176. *onh_t*; V^o_{nH}; $\mathring{A}^3$; H-free octant volumes ; steric extensive; -; -

177. *b_vol*; $V_{Bur.}$; $\mathring{A}^3$; Buried total volume; steric extensive; -; -

178. *b_vol_nh*; $S_{Bur.}$; $\mathring{A}^3$; H-free buried total volume; steric extensive; -; -

179. *bonh1*; $O^1_{Bur.}$; $\mathring{A}^3$; H-free buried $O(x, y, z)$ octant; steric extensive; -; -

180. *bonh2*; $O^2_{Bur.}$; $\mathring{A}^3$; H-free buried $O(-x, y, z)$ octant; steric extensive; -; -

181. *bonh3*; $O^3_{Bur.}$; $\mathring{A}^3$; H-free buried $O(-x, -y, z)$ octant; steric extensive; -; -

182. *bonh4*; $O^4_{Bur.}$; $\mathring{A}^3$; H-free buried $O(x, -y, z)$ octant; steric extensive; -; -

183. *bonh5*; $O^5_{Bur.}$; $\mathring{A}^3$; H-free buried $O(x, -y, -z)$ octant; steric extensive; -; -

184. *bonh6*; $O^6_{Bur.}$; $\mathring{A}^3$; H-free buried $O(-x, -y, -z)$ octant; steric extensive; -; -

185. *bonh7*; $O^7_{Bur.}$; $\mathring{A}^3$; H-free buried $O(-x, y, -z)$ octant; steric extensive; -; -

186. *bonh8*; $O^8_{Bur.}$; $\mathring{A}^3$; H-free buried $O(x, y, -z)$ octant; steric extensive; -; -

187. *bonhq1*; $Q^1_{Bur.}$; $\mathring{A}^3$; H-free buried $Q(x, y)$ quadrant; steric extensive; -; -

188. *bonhq2*; $Q^2_{Bur.}$; $\mathring{A}^3$; H-free buried $Q(-x, y)$ quadrant; steric extensive; -; -

189. *bonhq3*; $Q^3_{Bur.}$; $\mathring{A}^3$; H-free buried$Q(-x, -y)$ quadrant; steric extensive; -; -

190. *bonhq4*; $Q^4_{Bur.}$; $\mathring{A}^3$; H-free buried $Q(x, -y)$ quadrant; steric extensive; -; -

191. *bonhh1*; $H^1_{Bur.}$; $\mathring{A}^3$; H-free buried $H(x)$ half; steric extensive; -; -

192. *bonhh2*; $H^2_{Bur.}$; $\mathring{A}^3$; H-free buried $H(y)$ half; steric extensive; -; -

193. *bonhh3*; $H^3_{Bur.}$; $\mathring{A}^3$; H-free buried $H(z)$ half; steric extensive; -; -

194. *bonhh4*; $H^4_{Bur.}$; $\mathring{A}^3$; H-free buried $H(-x)$ half; steric extensive; -; -

195. *bonhh5*; $H^5_{Bur.}$; $\mathring{A}^3$; H-free buried $H(-y)$ half; steric extensive; -; -

196. *bonhh6*; $H^6_{Bur.}$; $\mathring{A}^3$; H-free buried $H(-z)$ half; steric extensive; -; -

197. *bonh_t*; $V^o_{Bur.}$; $\mathring{A}^3$; H-free buried octant volumes; steric extensive; -; -

198. *foq1*; $\hat{Q}^1$; -; Fraction of Q^1 to the total volume; steric intensive; -; -

199. *foq2*; $\hat{Q}^2$; -; Fraction of Q^2 to the total volume; steric intensive; -; -

200. *foq3*; $\hat{Q}^3$; -; Fraction of Q^3 to the total volume; steric intensive; -; -

201. *foq4*; $\hat{Q}^4$; -; Fraction of Q^4 to the total volume; steric intensive; -; -

202. *foh1*; $\hat{H}^1$; -; Fraction of H^1 to the total volume; steric intensive; -; -

203. *foh2*; $\hat{H}^2$; -; Fraction of H^2 to the total volume; steric intensive; -; -

204. *foh3*; $\hat{H}^3$; -; Fraction of H^3 to the total volume; steric intensive; -; -

205. *foh4*; $\hat{H}^4$; -; Fraction of H^4 to the total volume; steric intensive; -; -

206. *foh5*; $\hat{H}^5$; -; Fraction of H^5 to the total volume; steric intensive; -; -

207. *foh6*; $\hat{H}^6$; -; Fraction of H^6 to the total volume; steric intensive; -; -

208. *fonhq1*; $\hat{Q}^1_{nH}$; -; Fraction of Q^1_{nH} to the total volume; steric intensive; -; -

209. *fonhq2*; $\hat{Q}^2_{nH}$; -; Fraction of Q^2_{nH} to the total volume; steric intensive; -; -

210. *fonhq3*; $\hat{Q}^3_{nH}$; -; Fraction of Q^3_{nH} to the total volume; steric intensive; -; -

211. *fonhq4*; $\hat{Q}^4_{nH}$; -; Fraction of Q^4_{nH} to the total volume; steric intensive; -; -

212. *fonhh1*; $\hat{H}^1_{nH}$; -; Fraction of H^1_{nH} to the total volume; steric intensive; -; -

213. *fonhh2*; $\hat{H}^2_{nH}$; -; Fraction of H^2_{nH} to the total volume; steric intensive; -; -

214. *fonhh3*; $\hat{H}^3_{nH}$; -; Fraction of H^3_{nH} to the total volume; steric intensive; -; -

215. *fonhh4*; $\hat{H}^4_{nH}$; -; Fraction of H^4_{nH} to the total volume; steric intensive; -; -

216. *fonhh5*; $\hat{H}^5_{nH}$; -; Fraction of H^5_{nH} to the total volume; steric intensive; -; -

217. *fonhh6*; $\hat{H}^6_{nH}$; -; Fraction of H^6_{nH} to the total volume; steric intensive; -; -

218. *fbonhq1*; $\hat{Q}^1_{nH}$; -; Fraction of $Q^1_{Bur.}$ to the total volume; steric intensive; -; -

219. *fbonhq2*; $\hat{Q}^2_{nH}$; -; Fraction of $Q^2_{Bur.}$ to the total volume; steric intensive; -; -

220. *fbonhq3*; $\hat{Q}^3_{nH}$; -; Fraction of $Q^3_{Bur.}$ to the total volume; steric intensive; -; -

221. *fbonhq4*; $\hat{Q}^4_{nH}$; -; Fraction of $Q^4_{Bur.}$ to the total volume; steric intensive; -; -

222. *fbonhh1*; $\hat{H}^1_{nH}$; -; Fraction of $H^1_{Bur.}$ to the total volume; steric intensive; -; -

223. *fbonhh2*; $\hat{H}^2_{nH}$; -; Fraction of $H^2_{Bur.}$ to the total volume; steric intensive; -; -

224. *fbonhh3*; $\hat{H}^3_{nH}$; -; Fraction of $H^3_{Bur.}$ to the total volume; steric intensive; -; -

225. *fbonhh4*; $\hat{H}^4_{nH}$; -; Fraction of $H^4_{Bur.}$ to the total volume; steric intensive; -; -

226. *fbonhh5*; $\hat{H}^5_{nH}$; -; Fraction of $H^5_{Bur.}$ to the total volume; steric intensive; -; -

227. *fbonhh6*; $\hat{H}^6_{nH}$; -; Fraction of $H^6_{Bur.}$ to the total volume; steric intensive; -; -

228. *or*; G^R; -; Off diagonal volume ratio; steric intensive; Q^1 and Q^3 divided by Q^2 and Q^4; -

229. *onhr*; Q^R_{nH}; -; H-free off diagonal volume ratio; steric intensive; Q^1_{nH} and Q^3_{nH} divided by Q^2_{nH} and Q^4_{nH}; -

230. *bonhr*; $Q^{R}_{Bur.}$; -; H-free buried off diagonal volume ratio; steric intensive; $Q^{1}_{Bur.}$ and $Q^{3}_{Bur.}$ divided by $Q^{2}_{Bur.}$ and $Q^{4}_{Bur.}$; -

231. *pb_vol*; $\hat{V}^{H}_{Bur.}$; -; Fraction of total buried volume ; steric intensive; -; -

232. *pb_vol_nh*; $\hat{V}^{nH}_{Bur.}$; -; Fraction of total H-free buried volume ; steric intensive; -; -

S5 Linear models

S5.1 Inclusion of substrate effects

Currently, our pipeline does not include substrate features in the generated models, and therefore does not account for substrate effects which are key for evaluating substrate scope.

To include substrate features in our pipeline, a featurization strategy would have to be devised that can be applied generally to any substrate. This is challenging given the plausible diversity of substrates, but straightforward for single substrate families. As our goal is having a seamlessly transferable strategy that works for any reaction with bidentate ligands, we remain focused on catalyst optimization for now. This route will be explored in future work, as it is highly important to develop general catalysts.[31]

S5.2 Feature selection strategy

MLR models are constructed from a few selected features. Utilizing a brute-force approach (*i.e.*, evaluating every possible feature combination) in search for the best handful of features is time-consuming considering that our general featurization strategy (*vide supra*) yields 232 features. For these reasons, the forward step method in combination with Bayesian ridge regression (BRR) and leave-one-out cross validation (LOO) was used for feature selection (*vide infra*).[32] Note that BRR includes a regularization term, similar to the one found in ridge regression, that is used to penalize co-linearity and minimize overfitting. To further minimize overfitting and unnecessary model complexity, the maximum number of features

was set to three. This was deemed appropriate for models below 30 data points and would ideally account for one feature of each main family: electronic, steric, and topological. Note that in the case of the forward step method, models are allowed to possess multiple features belonging to the same family. We observed that by increasing the maximum number of features, by our current metrics, (adjusted R^2 of LOO samples) the best models would always possess the maximum amount of features, which would ultimately always lead to overfitted models. How different metric and/or cross validation schemes could help to solve these issues is currently being investigated.

When used in conjunction with Bayesian optimization, model construction is performed anew on every interaction. Thus, the feature selection above is re-run and may potentially yield different features as new data is acquired. This allows the MLR model to adapt to potential new trends in the underlying data and ensures that the interpretation is as accurate and relevant as possible at each step.

The forward step algorithm used for feature selection seeks to select one feature at a time to obtain a predictive model. The implementation used here[32] starts by testing all two feature combinations. Feature combinations with high collinearity (*e.g.*, R^2 of more than 0.5 between features in this case) are dropped. A given number of models with the largest R^2 against the target are kept and their R^2_{LOO} is computed. From this selection, all possible models with one more feature are generated. The collinearity between features is computed and feature combinations exceeding the collinearity criteria are once again dropped. R^2s against the target are computed again. In addition, models with uncommon features compared to the current top selection are also considered. The R^2_{LOO} is again computed for these candidates. Finally, one feature is removed from all saved models. If at some point one of these variations was considered but the R^2_{LOO} had not been computed before, that R^2_{LOO} is computed. This is followed by ranking, selection, and a final addition of models with uncommon features. The process is repeated until the desired amount of steps is reached.

Tables S3 to S6 show comparisons of different feature selection strategies: forward step,

KBest, recursive, nested,[3] and brute force. Except for the recursive method, the number of features was limited to 3. For the **OA** dataset, the brute force and nested approach both find the same model consisting of three topological features, in comparison, the model chosen by the forward step algorithm is more balanced as one of each class, electronic, steric and topological was selected. Similar observations are made for all other three datasets. We also note that the brute force and nested approach require between 4-6 days to compute in comparison to forward step which only takes about a minute.

Tables S8 to S11 show the top 10 models which were obtained with the aforementioned approach. Overall for **OA**, **CP**, and **DA**, variations are small and the most important features are present throughout the ranks. In the **CC** dataset, all features are changed from the first to second rank and the score changes significantly. Yet, most steric features describe the same region.

Table S3: Comparison of different feature selection strategies for OA. Leave-one-out (LOO) and leave-four-out (L4O) cross-validations are used. Mean average errors (MAE) are in kcal/mol.

**Method**	**OA**	**adj.** R^2_{LOO}	MAE_{LOO}	**adj.** R^2_{L4O}	MAE_{L4O}
Forward step	nbo1, fonhh4, f2kal	0.7077	0.1088	0.6643	0.1156
KBest	f2k	0.5589	0.1466	0.5471	0.1477
Recursive	s4chi, k_ups	-0.3992	0.2725	-0.3639	0.2664
Nested	2k, 3chi, Tnbo1	0.8646	0.0783	0.8561	0.0807
Brute force	2k, 3chi, Tnbo1	0.8646	0.0783	0.8561	0.0807

Table S4: Comparison of different feature selection strategies for CP. Leave-one-out (LOO) and leave-four-out (L4O) cross-validations are used. Mean average errors (MAE) are in kcal/mol.

**Method**	**CP**	**adj.** R^2_{LOO}	MAE_{LOO}	**adj.** R^2_{L4O}	MAE_{L4O}
Forward step	o8, fbonhq2, f2chi	0.7414	0.2399	0.7365	0.2431
KBest	bonhq2	0.5509	0.3402	0.5472	0.3411
Recursive	b_vol, b_vol_nh, o5, bonh_t, bonhq2, bonhh3, bonhh4, onhh1, oh3	0.3955	0.3318	0.3746	0.3379
Nested	zagreb2, 3chi, fbonhq2	0.7985	0.2211	0.7939	0.2243
Brute force	zagreb2, 3chi, fbonhq2	0.7985	0.2211	0.7939	0.2243

Table S5: **Comparison of different feature selection strategies for CC. Leave-one-out (LOO) and leave-four-out (L4O) cross-validations are used. Mean average errors (MAE) are in kcal/mol.**

**Method**	**CC**	**adj.** R^2_{LOO}	MAE_{LOO}	**adj.** R^2_{L4O}	MAE_{L4O}
Forward step	nbo-, fbonhq3, f1chi	0.6914	0.1435	0.6729	0.1502
KBest	o3, balaban	0.4168	0.2361	0.4088	0.2364
Recursive	zagreb1, 0k, b_vol, onh4, fT, k_upsb, bonhh3, bonhh6, onhh6	-0.6657	0.1966	-0.7691	0.206
Nested	onbo1, nbo-, fbonhq3	0.7230	0.1291	0.7093	0.1343
Brute force	onbo1, nbo-, fbonhq3	0.7230	0.1291	0.7093	0.1343

Table S6: **Comparison of different feature selection strategies for DA. Leave-one-out (LOO) and leave-four-out (L4O) cross-validations are used. Mean average errors (MAE) are in kcal/mol.**

**Method**	**DA**	**adj.** R^2_{LOO}	MAE_{LOO}	**adj.** R^2_{L4O}	MAE_{L4O}
Forward step	nbo-, bonh5, f3chiv	0.3009	0.2931	0.2869	0.2962
KBest	f3chiv	0.1354	0.3431	0.1269	0.344
Recursive	Tnbo1	-0.1489	0.3998	-0.1556	0.401
Nested	k_xia, 3chi, fzagreb1	0.4260	0.2571	0.4115	0.2619
Brute force	k_xia, 3chi, fzagreb1	0.4260	0.2571	0.4115	0.2619

Table S7: Out of sample predictions using different feature selections for OA

**Method**	80/20 MAE/RMSE	50/50 MAE/RMSE	IndaBOX MAE/RMSE
Forward step	0.05/0.07	0.15/0.18	0.15/0.17
KBest	0.08/0.09	0.17/0.21	0.07/0.08
Recursive	0.33/0.33	0.43/0.43	0.28/0.30
Nested	0.07/0.08	0.08/0.08	0.11/0.12
Brute force	0.07/0.08	0.08/0.08	0.11/0.12

Table S8: Top 10 models for OA with the BRR forward step approach.

**Rank**	**Features**	**adj.** R^2_{LOO}
1	nbo1, fonhh4, f2kal	0.7077
2	nbo1, fonhh1, f2kal	0.7077
3	nbo1, foh4, f2kal	0.7070
4	nbo1, foh1, f2kal	0.7070
5	bonh5, fbonhh4, f2k	0.7057
6	bonh5, fbonhh1, f2k	0.7057
7	nbo1, foh1, f2kab	0.7022
8	nbo1, foh4, f2kab	0.7022
9	nbo1, fonhh1, f2kb	0.7020
10	nbo1, fonhh4, f2kb	0.7020

Table S9: Top 10 models for CP with the BRR forward step approach.

Rank	Features	adj. R^2_{LOO}
1	o8, fbonhq2, f2chi	0.7414
2	onh8, fbonhq2, f2chi	0.7352
3	fbonhq2, crest_flex, fzagreb1	0.7310
4	fonhq1, fbonhq2, f2chi	0.7274
5	foq1, fbonhq2, f2chi	0.7264
6	bonh8, fbonhq2, f2chi	0.7257
7	fbonhq2, crest_flex, Tnbo1	0.7249
8	onr, fbonhq2, f2chi	0.7229
9	onhq1, fbonhq2, f2chi	0.7206
10	oq1, fbonhq2, f2chi	0.7203

Table S10: Top 10 models for CC with the BRR forward step approach.

Rank	Features	adj. R^2_{LOO}
1	nbo-, fbonhq3, f1chi	0.6914
2	onh3, f0chi, f3chiv	0.6284
3	oh2, balaban, f3chiv	0.6283
4	o3, f0chi, f3chiv	0.6280
5	onhh2, balaban, f3chiv	0.6240
6	o3, f1k, f3chiv	0.619598
7	nbo-, fbonhq3, f0chi	0.6189
8	onh3, f1k, f3chiv	0.6164
9	fbonhh5, f1kal, f3k	0.6161
10	fbonhh2, f1kal, f3k	0.6161

Table S11: Top 10 models for DA with the BRR forward step approach.

Rank	Features	adj. R^2_{LOO}
1	nbo-, bonh5, f3chiv	0.3009
2	bonh5, fonhq4, f3chiv	0.2901
3	bonh5, foq4, f3chiv	0.2898
4	onbo-, bonh5, f3chiv	0.2537
5	fbonhh3, f3chiv	0.2519
6	bonh5, f3chiv	0.2496
7	onbo1/2, bonh5, f3chiv	0.2382
8	bonh5, fbonhq2, f3chiv	0.2356
9	bonhh6, f3chiv	0.2344
10	onbo2, bonh5, f3chiv	0.2234

Table S12: Comparison of different cross-validation methods. 5-fold SD corresponds to the standard deviation of the 5-fold repetitions over 10 random runs. Scores in adjusted R^2 of left out samples.

Top model for	Features	LOO	LTO	5-fold	5-fold SD
OA with LOO	nbo1, fonhh4, f2kal	0.7077	0.6982	0.6737	0.0605
OA with LTO	nbo1, fonhh4, f2kal	0.7077	0.6982	0.6737	0.0605
OA with 5-Fold	angle, fonhq3, f2k	0.6996	0.6859	0.6783	0.0564
CP with LOO	o8, fbonhq2, f2chi	0.7414	0.7399	0.7350	0.0135
CP with LTO	o8, fbonhq2, f2chi	0.7414	0.7399	0.7350	0.0135
CP with 5-fold	o8, fbonhq2, f2chi	0.7414	0.7399	0.7350	0.0135
CC with LOO	nbo-, fbonhq3, f1chi	0.6914	0.6855	0.6321	0.0363
CC with LTO	nbo-, fbonhq3, f1chi	0.6914	0.6855	0.6321	0.0363
CC with 5-fold	onh3, f0chi, f3chiv	0.6284	0.6262	0.6368	0.0208
DA with LOO	nbo-, bonh5, f3chiv	0.3009	0.2968	0.1272	0.0848
DA with LTO	nbo-, bonh5, f3chiv	0.3009	0.2968	0.1272	0.0848
DA with 5-fold	fbonhh3, f3chiv	0.2519	0.2483	0.1906	0.0703

S5.3 Comparison of cross-validation methods

Different cross-validation strategies were tested to choose the best model and avoid over-fitting: leave-one-out (LOO), leave-two-out (LTO), and 5-fold. As shown in Table S12, the adjusted R^2 results are almost identical for most datasets and the same features are consistently selected. Although different models are usually identified with k-fold compared to LOO and LTO, the standard deviations of the repetitions (5-fold SD, 10 repetitions) fall in the range of the other models. Consequently, LOO was chosen as our default cross-validation strategy, as it is the least computationally expensive scheme.

The nested cross-validation strategy[3,33] was not demonstrated here due to its high computational cost with a large number of features (232 in our case).

S5.4 Extrapolation experiments

The ability of models to predict samples anticipated to have higher performance than those found in the training data is crucial. To ensure that our pipeline is capable of identifying such models, different data-splitting strategies were devised for the **OA** reaction (Figure S3). Across splits, we used Delaunay triangulation to determine whether samples from the

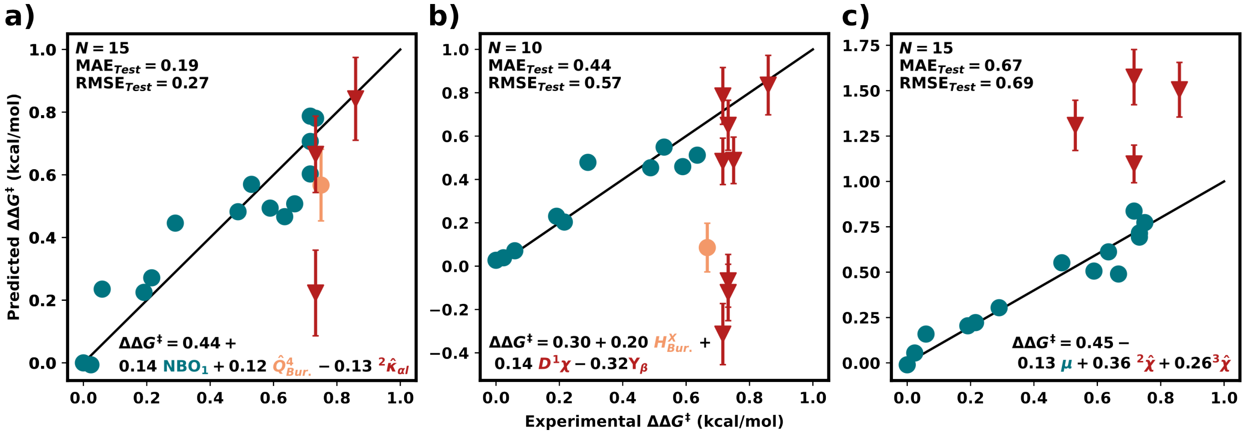

Figure S3: Three different test scenarios. Training points are represented by teal circles while test points are depicted as either orange circles (interpolations) or red triangles (extrapolations) with their corresponding 1σ standard deviation. (a) 80/20 train/test split of the top entries, with the lone-pair NBO energies of the smaller half, normalized buried volume south-east quadrant Q^4 $(x, -y)$, and normalized original Kier $^2\kappa_{\alpha l}$. (b) 50/50 train/test split of the top entries with buried volume of the larger half (x), the difference of 1-order χ indices $D^1\chi$, and extended bond Kier flexibility Υ_β. (c) IndaBOX ligands were left out of the training with dipole moment μ, normalized Hall $^2\chi$ index, and normalized Hall $^3\chi$ index.

test set are truly extrapolative (outside the convex hull for the selected feature subset, red triangles) or interpolative (orange circles).

In Figure S3a, the 80% of the data with the lowest $\Delta\Delta G^\ddagger$ was used as the training set to predict on the 20% with highest $\Delta\Delta G^\ddagger$ (as described in Section 3.2 and presented in Figure 4a of the main text). A single interpolative sample was found among the test set. In Figure S3b, the training set is composed of the 50% of samples with the lowest $\Delta\Delta G^\ddagger$, leading to a worse model (as expected due to training set reduction) where only one interpolative sample is found in the test set. Finally, in Figure S3c, the best performing ligand class (IndaBOX) was removed from the training to see how well the generated model would extrapolate to new and performant compound classes. The errors are large, and the predictions are far from the experimental values. Nevertheless, this model consistently overestimates IndaBOX ligands, which are, in all cases, true extrapolations. In all three cases, at least part of the test set is (correctly) predicted to outperform the training set, which in the BO setting would lead to subsequent experiments.

Additionally, this confirms that the data-splitting strategies considered here (based on $\Delta\Delta G^\ddagger$ and on ligand classes) lead to mostly extrapolative predictions.

In conclusion, these results show that extrapolation is a very challenging task, but as long as some predictions are qualitatively good, which we show to be the case in our examples, our strategy is usable in the context of Bayesian optimization, where in all cases appropriate ligands would be selected.

S5.5 Comparison with non-linear methods

For reference, we compared standard nonlinear regression methods such as Random forest regression (RFR) and Gaussian process regression (GPR) with our BRR pipeline using all 232 descriptors as features. RFR is an ensemble learning method built from a multitude of decision trees.[34] Advantages of RFR, besides being nonlinear, include the possibility of obtaining the importance of each feature used to train the model, as well as uncertainty estimates from the ensemble. RFR hyperparameters include the number of total estimators (10, 20, 50, 100, 200), splitting criterion (squared error, absolute, error, Friedman mean squared error, Poisson), and maximum depth of the trees (1, 2, 5, None), all of which were optimized in a LOO cross-validated grid search approach. Finally, GPR is a nonlinear kernel-based method.[35] Its Bayesian approach to learning also allows the estimation of prediction uncertainty and is the most popular regression method for BO. The radial basis function (RBF) kernel was used, and its hyperparameters were optimized by maximizing the log marginal likelihood (the standard in GPR). Other kernel functions were tested (including Linear and Matern kernels) but did not yield any significant improvements over the RBF kernel. The Sklearn package[36] was used for BRR and RFR, and GPy for the GPR.[37]

In Figure S4, the three feature BRR models from main text Figure 3 are compared with nonlinear ML regression methods, RFR and GPR, which were trained with all 232 available features. With small datasets like the ones used in this work, such nonlinear methods tend to overfit, which increases the LOO CV error significantly (*i.e.*, the models fit the training data too well and are not able to generalize to unseen cases). The BRR models have lower LOO MAE and standard deviations for all four datasets, and are therefore more suited than

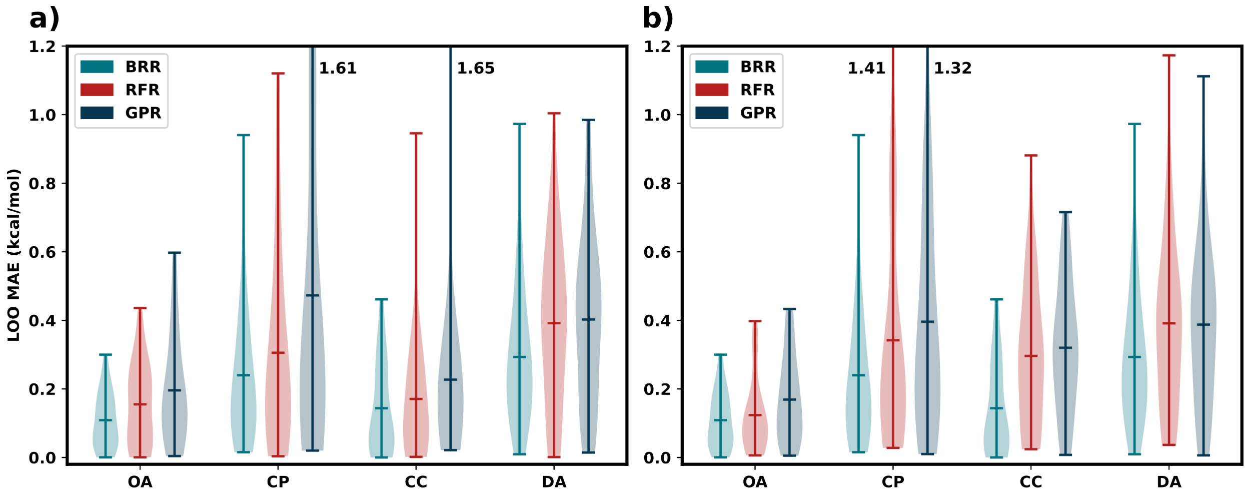

Figure S4: Comparison of the three-feature Bayesian ridge regression (BRR, our approach) models with random forest regression (RFR) and Gaussian process regression (GPR) models. Large outlier bounds are indicated numerically. The y-axis describes the leave-one-out (LOO) mean absolute error (MAE). (a) No feature selection was performed on RFR and GPR. (b) KBest (k=3) feature selection was done for RFR and GPR.

nonlinear methods in this low data regime. This is most noticeably observed for noisy data, such as it is the case in **DA**. The generality of the **DA** BRR model makes it less prone to over- or underestimation. In terms of interpretability, the feature importances of RFR were found to be systematically different from the BRR models (Table S13). Of the top 10 most important features of RFR, no match with the BRR model features for **OA**, **CP**, and **CC** sets was found. Only for the **DA**, where the normalized $^3\chi_v$ index was found to be the second most important feature overall. GPR is not trivial to interpret, and thus would require the use of Shapley values.[38]

Table S13: Top 10 feature importances given by random forest regression (RFR) model for each dataset.

**Rank**	**OA**	**CP**	**CC**	**DA**
1.	f1k	f0chiv	o2	f2chiv
2.	f1kal	f1chi	onh2	**f3chiv**
3.	f1kb	bonhh3	bonhh5	nbo1
4.	fhosoya	fbonhq1	t_ova_nh	f1kb
5.	f1chi	onh1	bonh7	f1chi
6.	lumo	f3k	bonhh3	nbo2
7.	fbonhq3	bonhq4	f2chiv	onh8
8.	fonhh1	o1	bonh_t	cnbo1
9.	kier_al	t_ova_nh	b_vol	k_ups
10.	f1ka	fkier_b	onbo1	nbo+

References

(1) Hari, D. P.; Waser, J. Enantioselective Copper-Catalyzed Oxy-Alkynylation of Diazo Compounds. *J. Am. Chem. Soc.* **2017**, *139*, 8420–8423.

(2) Aguado-Ullate, S.; Urbano-Cuadrado, M.; Villalba, I.; Pires, E.; García, J. I.; Bo, C.; Carbó, J. J. Predicting the Enantioselectivity of the Copper-Catalysed Cyclopropanation of Alkenes by Using Quantitative Quadrant-Diagram Representations of the Catalysts. *Chem. Eur. J.* **2012**, *18*, 14026–14036.

(3) Lau, S. H.; Borden, M. A.; Steiman, T. J.; Wang, L. S.; Parasram, M.; Doyle, A. G. Ni/Photoredox-Catalyzed Enantioselective Cross-Electrophile Coupling of Styrene Oxides with Aryl Iodides. *J. Am. Chem. Soc.* **2021**, *143*, 15873–15881.

(4) Evans, D. A.; Lectka, T.; Miller, S. J. Bis(imine)-copper(II) complexes as chiral lewis acid catalysts for the Diels-Alder reaction. *Tetrahedron Lett.* **1993**, *34*, 7027–7030.

(5) Davies, I. W.; Gerena, L.; Castonguay, L.; Senanayake, C. H.; Larsen, R. D.; Verhoeven, T. R.; Reider, P. J. The influence of ligand bite angle on the enantioselectivity of copper(II)-catalysed Diels-Alder reactions. *Chem. Commun.* **1996**, 1753–1754.

(6) Ghosh, A. K.; Mathivanan, P.; Cappiello, J. Conformationally constrained bis(oxazoline) derived chiral catalyst: A highly effective enantioselective Diels-Alder reaction. *Tetrahedron Lett.* **1996**, *37*, 3815–3818.

(7) Davies, I. W.; Gerena, L.; Cai, D.; Larsen, R. D.; Verhoeven, T. R.; Reider, P. J. A conformational toolbox of oxazoline ligands. *Tetrahedron Lett.* **1997**, *38*, 1145–1148.

(8) Evans, D. A.; Miller, S. J.; Lectka, T.; von Matt, P. Chiral Bis(oxazoline)copper(II) Complexes as Lewis Acid Catalysts for the Enantioselective Diels-Alder Reaction. *J. Am. Chem. Soc.* **1999**, *121*, 7559–7573.

(9) Kanemasa, S.; Adachi, K.; Yamamoto, H.; Wada, E. Bisoxazoline and Bioxazoline Chiral Ligands Bearing 4-Diphenylmethyl Shielding Substituents. Diels-Alder Reaction of Cyclopentadiene with 3-Acryloyl-2-oxazolidinone Catalyzed by the Aqua Nickel(II) Complex. *Bull. Chem. Soc. Jpn.* **2000**, *73*, 681–687.

(10) O'Leary, P.; Krosveld, N. P.; De Jong, K. P.; van Koten, G.; Klein Gebbink, R. J. Facile and rapid immobilization of copper(II) bis(oxazoline) catalysts on silica: application to Diels-Alder reactions, recycling, and unexpected effects on enantioselectivity. *Tetrahedron Lett.* **2004**, *45*, 3177–3180.

(11) Xiong, H.; Xu, H.; Liao, S.; Xie, Z.; Tang, Y. Copper-Catalyzed Highly Enantioselective Cyclopentannulation of Indoles with Donor–Acceptor Cyclopropanes. *J. Am. Chem. Soc.* **2013**, *135*, 7851–7854.

(12) Meng, J.-c.; Fokin, V. V.; Finn, M. Kinetic resolution by copper-catalyzed azide–alkyne cycloaddition. *Tetrahedron Lett.* **2005**, *46*, 4543–4546.

(13) Faita, G.; Livieri, A. *Encyclopedia of Reagents for Organic Synthesis*; John Wiley & Sons, Ltd: Chichester, UK, 2012.

(14) Park, J. K.; Kim, S.-W.; Hyeon, T.; Kim, B. Heterogeneous asymmetric Diels–Alder reactions using a copper–chiral bis(oxazoline) complex immobilized on mesoporous silica. *Tetrahedron Asymmetry* **2001**, *12*, 2931–2935.

(15) Thirupathi, N.; Wei, F.; Tung, C.-H.; Xu, Z. Divergent synthesis of chiral cyclic azides via asymmetric cycloaddition reactions of vinyl azides. *Nat. Commun.* **2019**, *10*, 3158.

(16) Kier, L. B. An Index of Molecular Flexibility from Kappa Shape Attributes. *Quant. Struct.-Act. Relat.* **1989**, *8*, 221–224.

(17) Caron, G.; Digiesi, V.; Solaro, S.; Ermondi, G. Flexibility in early drug discovery: focus on the beyond-Rule-of-5 chemical space. *Drug Discov. Today* **2020**, *25*, 621–627.

(18) Kier, L. B. A Shape Index from Molecular Graphs. *Quant. Struct.-Act. Relat.* **1985**, *4*, 109–116.

(19) Kier, L. B. Shape Indexes of Orders One and Three from Molecular Graphs. *Quant. Struct.-Act. Relat.* **1986**, *5*, 1–7.

(20) Kier, L. B. Distinguishing Atom Differences in a Molecular Graph Shape Index. *Quant. Struct.-Act. Relat.* **1986**, *5*, 7–12.

(21) Hall, L. H.; Kier, L. B. Determination of Topological Equivalence in Molecular Graphs from the Topological State. *Quant. Struct.-Act. Relat.* **1990**, *9*, 115–131.

(22) Kier, L. B.; Hall, L. H. A Differential Molecular Connectivity Index. *Quant. Struct.-Act. Relat.* **1991**, *10*, 134–140.

(23) Hall, L. H.; Mohney, B.; Kier, L. B. The Electrotopological State: An Atom Index for QSAR. *Quant. Struct.-Act. Relat.* **1991**, *10*, 43–51.

(24) Fisanick, W.; Cross, K. P.; Rusinko, A. Characteristics of computer-generated 3D and related molecular property data for CAS registry substances. *Tetrahedron Comput. Methodol.* **1990**, *3*, 635–652.

(25) Pracht, P.; Bohle, F.; Grimme, S. Automated exploration of the low-energy chemical space with fast quantum chemical methods. *Phys. Chem. Chem. Phys.* **2020**, *22*, 7169–7192.

(26) Wiener, H. Structural Determination of Paraffin Boiling Points. *J. Am. Chem. Soc.* **1947**, *69*, 17–20.

(27) Hosoya, H. Topological Index. A Newly Proposed Quantity Characterizing the Topological Nature of Structural Isomers of Saturated Hydrocarbons. *Bull. Chem. Soc. Jpn.* **1971**, *44*, 2332–2339.

(28) Balaban, A. T. Highly discriminating distance-based topological index. *Chem. Phys. Lett.* **1982**, *89*, 399–404.

(29) Wicker, J. G. P.; Cooper, R. I. Beyond Rotatable Bond Counts: Capturing 3D Conformational Flexibility in a Single Descriptor. *J. Chem. Inf. Model.* **2016**, *56*, 2347–2352.

(30) Cammarota, R. C.; Liu, W.; Bacsa, J.; Davies, H. M. L.; Sigman, M. S. Mechanistically Guided Workflow for Relating Complex Reactive Site Topologies to Catalyst Performance in C–H Functionalization Reactions. *J. Am. Chem. Soc.* **2022**, *144*, 1881–1898.

(31) Gallarati, S.; van Gerwen, P.; Laplaza, R.; Brey, L.; Makaveev, A.; Corminboeuf, C. A genetic optimization strategy with generality in asymmetric organocatalysis as a primary target. *Chem. Sci.* **2024**, *15*, 3640–3660.

(32) Clements, H. D.; Flynn, A. R.; Nicholls, B. T.; Grosheva, D.; Lefave, S. J.; Merriman, M. T.; Hyster, T. K.; Sigman, M. S. Using Data Science for Mechanistic Insights and Selectivity Predictions in a Non-Natural Biocatalytic Reaction. *J. Am. Chem. Soc.* **2023**, *145*, 17656–17664.

(33) Żurański, A. M.; Martinez Alvarado, J. I.; Shields, B. J.; Doyle, A. G. Predicting Reaction Yields via Supervised Learning. *Acc. Chem. Res.* **2021**, *54*, 1856–1865.

(34) Breiman, L. Random Forests. *Mach. Learn.* **2001**, *45*, 5–32.

(35) Rasmussen, C. E.; Williams, C. K. I. *Gaussian Processes for Machine Learning (Adaptive Computation and Machine Learning)*; The MIT Press, 2005.

(36) Pedregosa, F.; Varoquaux, G.; Gramfort, A.; Michel, V.; Thirion, B.; Grisel, O.; Blondel, M.; Prettenhofer, P.; Weiss, R.; Dubourg, V. et al. Scikit-learn: Machine Learning in Python. *J. Mach. Learn. Res.* **2011**, *12*, 2825–2830.

(37) GPy GPy: A Gaussian process framework in python. http://github.com/SheffieldML/GPy, 2012; (accessed 2024-05-15).

(38) Lundberg, S. M.; Lee, S.-I. In *Advances in Neural Information Processing Systems 30*; Guyon, I., Luxburg, U. V., Bengio, S., Wallach, H., Fergus, R., Vishwanathan, S., Garnett, R., Eds.; Curran Associates, Inc., 2017; pp 4765–4774.
